# Distinct biological signature and modifiable risk factors underlie the comorbidity between major depressive disorder and cardiovascular disease

**DOI:** 10.1038/s44161-024-00488-y

**Published:** 2024-06-17

**Authors:** Jacob Bergstedt, Joëlle A. Pasman, Ziyan Ma, Arvid Harder, Shuyang Yao, Nadine Parker, Jorien L. Treur, Dirk J. A. Smit, Oleksandr Frei, Alexey A. Shadrin, Joeri J. Meijsen, Qing Shen, Sara Hägg, Per Tornvall, Alfonso Buil, Thomas Werge, Jens Hjerling-Leffler, Thomas D. Als, Anders D. Børglum, Cathryn M. Lewis, Andrew M. McIntosh, Unnur A. Valdimarsdóttir, Ole A. Andreassen, Patrick F. Sullivan, Yi Lu, Fang Fang

**Affiliations:** 1 Unit of Integrative Epidemiology, Institute of Environmental Medicine, Karolinska Institutet, Stockholm, Sweden; 2 Department of Medical Epidemiology and Biostatistics, Karolinska Institutet, Stockholm, Sweden; 3 Department Medical Biochemistry and Biophysics, Karolinska Institutet, Stockholm, Sweden; 4 Centre for Precision Psychiatry, Division of Mental Health and Addiction, University of Oslo and Oslo University Hospital, Oslo, Norway; 5 Genetic Epidemiology, Department of Psychiatry, Amsterdam UMC, University of Amsterdam, Amsterdam, the Netherlands; 6 Centre for Bioinformatics, Department of Informatics, University of Oslo, Oslo, Norway; 7 K.G. Jebsen Centre for Neurodevelopmental Disorders, University of Oslo and Oslo University Hospital, Oslo, Norway; 8 Institute of Biological Psychiatry, Mental Health Center Sct. Hans, Mental Health Services Copenhagen, Roskilde, Denmark; 9 Clinical Research Center for Mental Disorders, Shanghai Pudong New Area Mental Health Center, Tongji University School of Medicine, Shanghai, China; 10 Institute for Advanced Study, Tongji University, Shanghai, China; 11 Department of Clinical Science and Education Södersjukhuset, Karolinska Institutet, Stockholm, Sweden; 12 Department of Clinical Medicine, Faculty of Health and Medical Sciences, University of Copenhagen, Copenhagen, Denmark; 13 Department of Molecular Medicine (MOMA), Molecular Diagnostic Laboratory, Aarhus University Hospital, Aarhus, Denmark; 14 Department of Biomedicine, Aarhus University, Aarhus, Denmark; 15 Center for Genomics and Personalized Medicine, Aarhus, Denmark; 16 Social, Genetic and Developmental Psychiatry Centre, King’s College London, London, UK; 17 Department of Medical and Molecular Genetics, King’s College London, London, UK; 18 Centre for Clinical Brain Sciences, University of Edinburgh, Royal Edinburgh Hospital, Edinburgh, UK; 19 Centre for Genomics and Experimental Medicine, University of Edinburgh, Edinburgh, UK; 20 Centre of Public Health Sciences, Faculty of Medicine, School of Health Sciences, University of Iceland, Reykjavik, Iceland; 21 Department of Epidemiology, Harvard TH Chan School of Public Health, Harvard University, Boston, MA USA; 22 Department of Psychiatry, University of North Carolina at Chapel Hill, Chapel Hill, NC USA; 23 Department of Genetics, University of North Carolina at Chapel Hill, Chapel Hill, NC USA

**Keywords:** Genome-wide association studies, Cardiovascular diseases, Genetics research

## Abstract

Major depressive disorder (MDD) and cardiovascular disease (CVD) are often comorbid, resulting in excess morbidity and mortality. Here we show that CVDs share most of their genetic risk factors with MDD. Multivariate genome-wide association analysis of shared genetic liability between MDD and atherosclerotic CVD revealed seven loci and distinct patterns of tissue and brain cell-type enrichments, suggesting the involvement of the thalamus. Part of the genetic overlap was explained by shared inflammatory, metabolic and psychosocial or lifestyle risk factors. Our data indicated causal effects of genetic liability to MDD on CVD risk, but not from most CVDs to MDD, and showed that the causal effects were partly explained by metabolic and psychosocial or lifestyle factors. The distinct signature of MDD–atherosclerotic CVD comorbidity suggests an immunometabolic subtype of MDD that is more strongly associated with CVD than overall MDD. In summary, we identified biological mechanisms underlying MDD–CVD comorbidity and modifiable risk factors for prevention of CVD in individuals with MDD.

## Abstract (web summary)

Bergstedt et al. show that the effects of genetic liability to major depressive disorder can cause an increase in cardiovascular risk and that metabolic, psychological and lifestyle factors are partly responsible for this association.

## Main

Major depressive disorder (MDD) and cardiovascular disease (CVD) are comorbid^1,2^. Several mechanisms might explain the observed comorbidity^2^. One explanation is that genetic risk factors for MDD and CVDs overlap^3,4^. While observed genetic correlations between MDD and CVD are modest^2–4^, this may reflect local genetic correlations of opposing directions attenuating correlation on the genome-wide level, leading to an underestimation of the genetic overlap^5^. The large polygenicity of MDD^6^ might also mask subtypes with stronger genetic relationships to CVD.

The observed MDD–CVD comorbidity could also be due to nongenetic factors^7^. Cardiovascular risk factors such as high systolic blood pressure, high body mass index (BMI), high levels of low-density lipoprotein cholesterol, high levels of physical inactivity, presence of type II diabetes and smoking have all been associated with MDD^8–10^. Moreover, accumulating data show that psychosocial and/or lifestyle factors associated with MDD, such as low educational attainment, exposure to childhood maltreatment, loneliness and atypical sleep patterns, are also important risk factors for CVD^11–14^.

One common mechanism underlying MDD and CVD, as well as their shared risk factors, could be chronic inflammation. Atherosclerosis, the accumulation of fibrofatty lesions in the arterial wall, is the main cause of CVD^15^. The buildup of atherosclerotic plaque is a long-term inflammatory process mediated by immune components in crosstalk with arterial wall cells^16^. Many lines of evidence also support a role for inflammation in MDD^17^. Excessive or long-term psychosocial stress promote the maturation and release of inflammatory cytokines such as interleukin (IL)6, which activate the central nervous system to produce behaviors related to MDD^17^. Importantly, low-grade inflammation, measured by high C-reactive protein levels, has been observed in more than a quarter of patients with depression^18^, suggesting the presence of an inflammatory subtype of MDD^19^, which might be especially strongly associated with CVD.

The full extent of the genetic overlap between MDD and CVD has not been explored. It remains unknown whether the genetic overlap is associated with specific tissues or brain cell types, or how this overlap relates to shared risk factors such as blood pressure, psychosocial or lifestyle traits, metabolic traits, and inflammation. Moreover, causal effects linking these traits are not fully understood^20–23^.

In this Article, we dissect the genetic overlap between MDD and CVD (peripheral artery disease, heart failure, coronary artery disease, stroke and atrial fibrillation). First, we assessed genetic overlap between MDD and CVD on the genome-wide level, as well as on the level of local partitions of the genome and overlapping risk variants with MiXeR^24^ and Local Analysis of (co)Variant Association (LAVA)^25^. Second, we identified genetic variants and genes that contribute to the shared genetic liability between MDD and atherosclerotic CVD (ASCVD; peripheral artery disease, heart failure, coronary artery disease and stroke) using genomic structural equation modeling (SEM)^26^. We mapped identified variants to brain cell types using annotations based on recent single-cell RNA sequencing in postmortem human brain samples^27^. Third, we assessed shared risk factors explaining the genetic correlation between MDD and CVD. Finally, we used Mendelian randomization (MR) to investigate putative causal pathways linking MDD and CVD.

## Results

### Study design

**Fig. 1:**
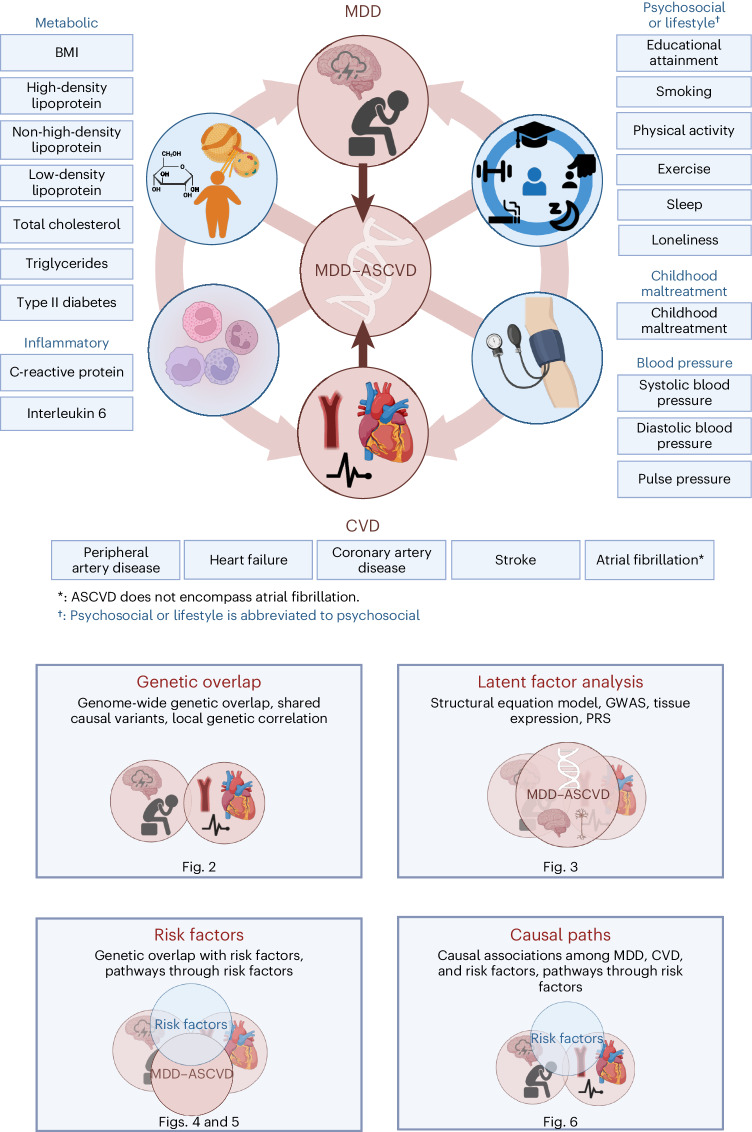
Study design. The comorbidity between MDD and CVD was investigated using genetic and causal inference methods, including assessing overlap with and mediation through shared risk factors (blood pressure, psychosocial or lifestyle, childhood maltreatment, metabolic and inflammation; Supplementary Table 1). The risk factor group psychosocial or lifestyle is abbreviated to psychosocial. Created with BioRender.com (license agreements VG26BG3VTL, MK26BG46H3).

We acquired summary statistics from the largest and most recent genome-wide association studies (GWASs) so far of MDD^6^, MDD symptoms, CVDs and shared risk factors (Fig. 1 and Supplementary Table 1). We used summary statistics from a GWAS of MDD involving 294,322 cases^6^. For CVDs, we used summary statistics from GWASs of peripheral artery disease^28^, heart failure^29^, coronary artery disease^30^, stroke^31^ and atrial fibrillation^32^, based on 12,086, 47,304, 181,523, 73,652 and 60,620 cases, respectively. We considered five categories of risk factors: blood pressure^33^, psychosocial or lifestyle^34–38^, childhood maltreatment^39^, metabolic^40–42^ and inflammation^43,44^. These data were used together with a comprehensive set of methods to elucidate etiological pathways underlying the comorbidity between MDD and CVD (Fig. 1).

### Most genetic risk factors for CVD overlap with MDD

**Extended Data Fig. 1:**
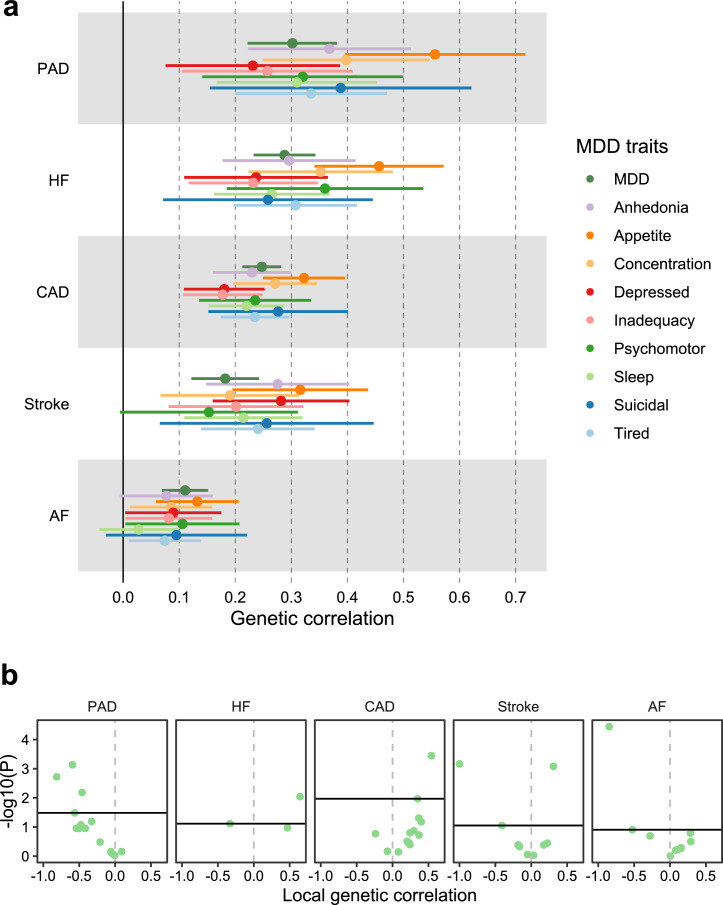
Genetic correlation of MDD and MDD symptoms with CVDs. **a**, Results are based on LD Score Regression analysis. Points and error bars represent mean genetic correlation and 95% CIs. **b**, Local genetic correlation between MDD and CVDs in 16 loci in the HLA region. Only loci with marginally statistically significant local heritability for both traits are shown. Points above the vertical line are significant based on multiple testing adjustment for considered loci performed for each CVD trait separately. Multiple testing was adjusted for using the Benjamini-Hochberg procedure. Empirical *P*-values were obtained via a permutation procedure with partial integration, evaluating the two-sided hypothesis of no association using the estimated parameters as test statistics **a-b** Sample sizes for underlying GWAS summary statistics are reported in Supplementary Table 1. AF=Atrial Fibrillation; CAD=Coronary Artery Disease; HF=Heart Failure; MDD=Major depressive disorder; PAD=Peripheral Artery Disease. Source data

**Extended Data Fig. 2:**
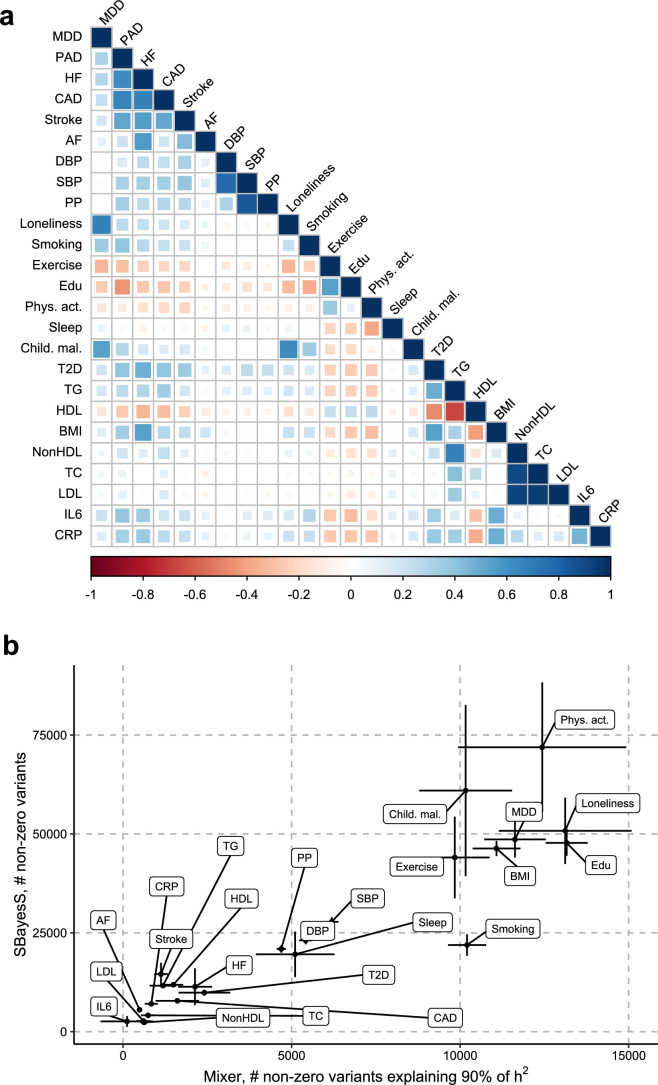
Polygenicity and genetic correlation for MDD, CVD, and risk factors. **a**, Heatmap of the genetic correlations between MDD, the CVDs, and the risk factors, with the color indicating the effect direction (negative: red, positive: blue) and the size and shade of the square illustrating the size of the correlation. Results are based on LD Score Regression analysis. **b**, Polygenicity estimates from SBayesS (y-axis) and MiXeR (x-axis) for MDD, the CVDs, and the risk factors. Note that for PAD polygenicity estimates did not converge for SBayeS, possibly because of the few number of cases. Points and error bars represent the mean number of estimated non-zero variants and 95% CIs. **a, b** Sample sizes for underlying GWAS summary statistics are reported in Supplementary Table 1. AF=Atrial Fibrillation; CAD=Coronary Artery Disease; Child. Mal.=Childhood Maltreatment; CRP=C-Reactive Protein; DBP=Diastolic Blood Pressure; Edu=Educational attainment; HDL=High-Density Lipoprotein; HF=Heart Failure; IL6=Interleukin-6; LDL=Low-Density Lipoprotein; MDD=Major Depressive Disorder; NonHDL=Non-High-Density Lipoprotein; PAD=Peripheral Artery Disease; Phys. Act.=Physical activity; PP=Pulse Pressure; Psychosocial=Psychosocial/lifestyle; SBP=Systolic Blood Pressure; T2D=Type II Diabetes; TC=Total Cholesterol; TG=Triglycerides. Source data

We found moderate genetic correlations between MDD and CVDs, as estimated with linkage disequilibrium score regression (LDSC^45^; Extended Data Fig. 1 and Supplementary Table 2). The strongest correlations were noted for peripheral artery disease (*r*_*g*_ = 0.30, 95% CI [0.22, 0.38], *P* = 1 × 10^−13^), heart failure (*r*_*g*_ = 0.29, 95% CI [0.23, 0.34], *P* = 1 × 10^−24^) and coronary artery disease (*r*_*g*_ = 0.25, 95% CI [0.21, 0.28], *P* = 9 × 10^−45^), while smaller but statistically significant correlations were observed for stroke and atrial fibrillation (*r*_*g*_ = 0.18 and *r*_*g*_ = 0.11, *P* < 1 × 10^−7^). ASCVDs showed strong correlations among each other while atrial fibrillation was only moderately genetically correlated to the ASCVDs (Extended Data Fig. 2a and Supplementary Table 2). For MDD symptoms, poor appetite or overeating showed consistently the strongest genetic correlations with the CVDs (Extended Data Fig. 1a and Supplementary Table 3).

**Fig. 2:**
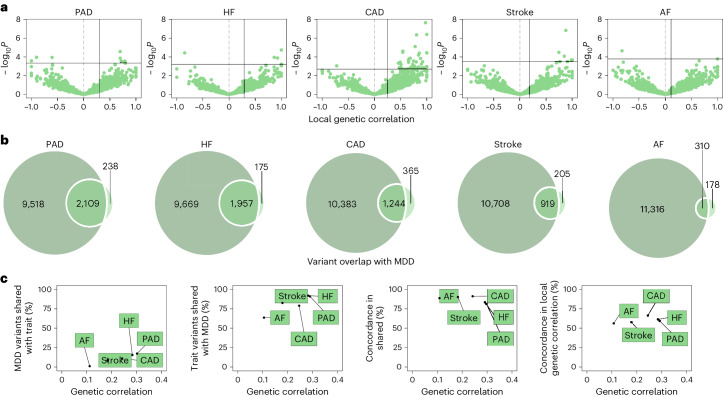
Genetic overlap between MDD and CVD beyond genome-wide genetic correlation. **a**, Volcano plots based on LAVA results showing genomic loci (green dots) with the local genetic correlation between MDD and each of the CVDs (*x* axis) and the corresponding *P* value (*y* axis). Loci exceeding the horizontal line are significant at *P*_FDR_ < 0.05 (Benjamini–Hochberg-adjusted *P* value). Multiple testing was performed separately for each trait over all considered loci. Empirical *P* values were obtained via a permutation procedure with partial integration, evaluating the two-sided hypothesis of no association using the estimated parameters as test statistics. **b**, Venn diagrams based on MiXeR results showing the number of causal variants (number of nonzero variants required to explain 90% of trait heritability) that are unique to MDD (left circle), unique to CVD (nonoverlapping part of right circle) or shared between MDD and CVD (overlapping part of circles). **c**, Genetic correlation estimated by LDSC (*x* axis) against the percentage of MDD causal variants that are shared with the CVD trait as estimated by MiXeR (first plot), the percentage of CVD trait causal variants that are shared with MDD (second plot) and the percentage of shared variants that have concordant effect directions (third plot). The fourth plot shows the percentage of local genetic correlations from LAVA that have concordant effect directions on the *y* axis. In **a**–**c**, the sample sizes and information for underlying summary statistics GWASs are reported in Supplementary Table 1. AF, atrial fibrillation; CAD, coronary artery disease; HF, heart failure; PAD, peripheral artery disease. Source data

We estimated local genetic correlations between MDD and CVDs in each of 2,495 distinct genomic regions using LAVA^25^. We found 54 statistically significant, predominantly positive, local correlations between MDD and CVDs, 40 of which were noted for MDD and coronary artery disease (Fig. 2 and Supplementary Table 4). We also assessed correlations between MDD and the CVDs in 16 loci in the human leukocyte antigen (HLA) region (Extended Data Fig. 1b). Out of 50 assessed correlations, 8 were statistically significant, indicating that this region is a hotspot of correlation between MDD and CVDs.

Next, we investigated genetic overlap on the level of genetic variants using MiXeR^24^ (Supplementary Tables 5 and 6). We identified more causal variants, that is, genetic variants with nonzero effects taking linkage disequilibrium (LD) into account, for MDD than for the CVDs, suggesting that MDD is more polygenic than CVD. To verify these results, we estimated polygenicity using a complementary approach implemented in SBayesS^46^, which showed estimates that were highly correlated with those of MiXeR (Extended Data Fig. 2b and Supplementary Table 7).

Bivariate MiXeR results showed that CVDs shared most of their causal variants with MDD (from 64% for atrial fibrillation to 92% for heart failure; Fig. 2b and Supplementary Table 6) whereas MDD shared only few of its causal variants with CVDs (<20%). Performance metrics indicated that results for peripheral artery disease should be interpreted with caution. Both shared genetic variants and local genetic correlations exhibited strong degrees of effect direction concordance (Fig. 2c), suggesting that genetic risk variants for CVDs are strongly correlated with a genetic subcomponent of MDD.

### Shared genetic liability to MDD and CVD

**Fig. 3:**
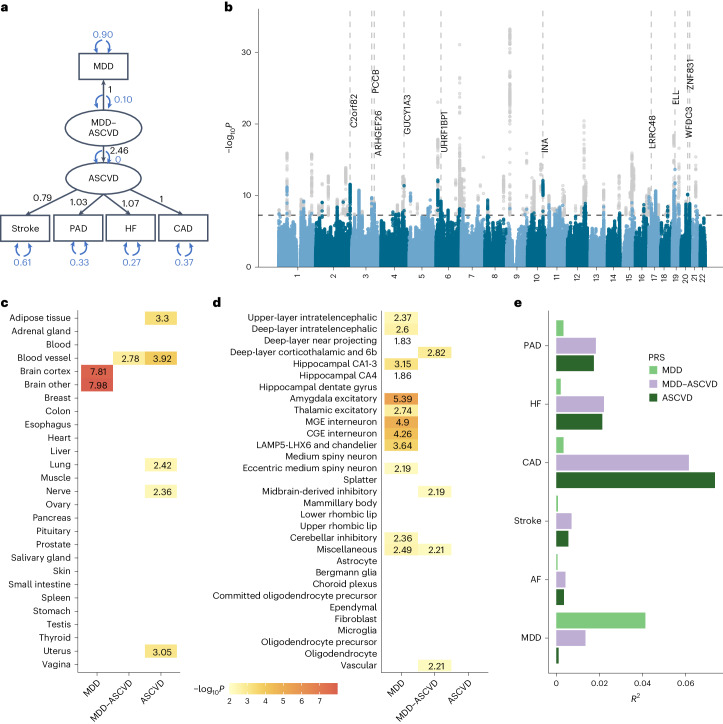
Shared genetic liability latent factor for MDD–ASCVD. **a**, The latent factor model as specified in genomic SEM with the ‘observed’ variables in rectangles and the latent variables in circles. Factor loadings (standardized with respect to the genetic variance of the traits) are given in black and variances in blue. **b**, Latent MDD–ASCVD factor GWAS results. The *x* axis shows the genomic position and the *y* axis shows statistical significance as −log_10_*P*. Genome-wide significant SNPs (*P* *<* 5 × 10^−8^) that were filtered out because of significant heterogeneity *Q*_SNP_ are displayed in gray. The top ten eQTL genes are displayed with dashed vertical lines indicating their position. *P* values were computed using a two-sided *Z*-test. **c**, Enrichment results in GTEx tissues for the latent MDD–ASCVD factor, with latent ASCVD (without MDD) and MDD only as comparison. **d**, Enrichment results for the latent MDD–ASCVD factor, latent ASCVD and MDD only in brain cell types. **e**, The proportion of variance explained in MDD and CVD phenotypes in the UKB (defined using ICD codes listed in Supplementary Table 14) by each of three PRSs for the latent MDD–ASCVD factor, latent ASCVD or MDD only. **c**,**d**, Enrichment is measured using significance testing in a one-sided *Z*-test displayed as −log_10_(*P*). Only tissues with a significant association (*P*_FDR_ < 0.05; Benjamini–Hochberg adjustment for multiple testing) are shown. Multiple testing was performed over tested tissues/cell-types. In **a**–**e**, sample sizes and information for underlying GWAS summary statistics are reported in Supplementary Table 1. MDD–ASCVD, common factor for MDD and ASCVD; ASCVD, common factor for the atherosclerotic cardiovascular diseases. CA, cornu ammonis; CGE, caudal ganglionic eminence; MGE, medial ganglionic eminence. Source data

**Extended Data Fig. 3:**
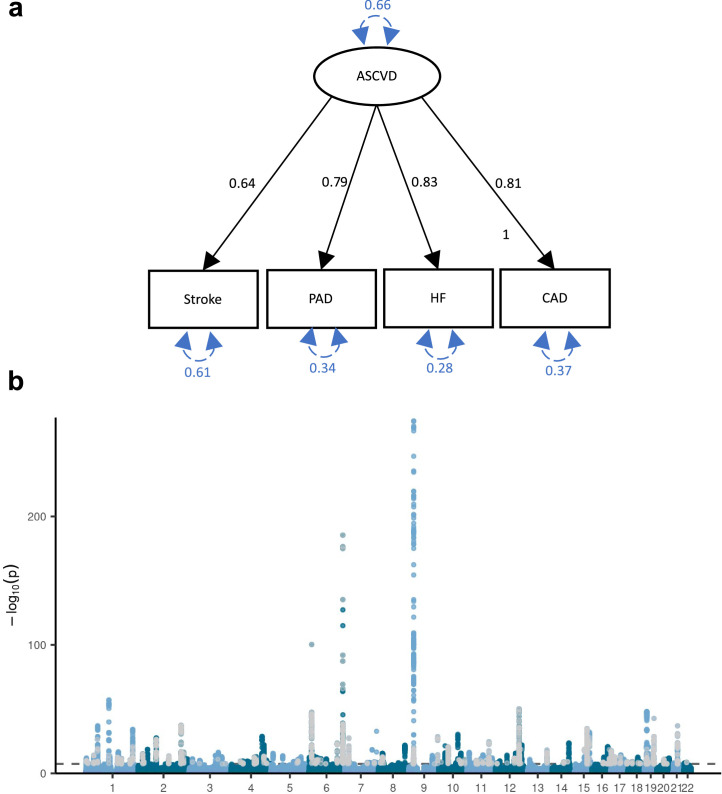
Genetic liability to ASCVD latent factor. **a**, Latent atherosclerotic CVD (ASCVD) model, defined by stroke, peripheral artery disease (PAD), heart failure (HF), and coronary artery disease (CAD). All ‘observed’ traits are based on GWAS summary statistics. Results are from confirmatory factor analysis in Genomic SEM, and standardized factor loadings are given for each path. Circular dashed arrows give the trait variance. **b**, Manhattan plot of the GWAS on ASCVD, with each dot representing a SNP with its position on the x-axis and its *P-*value on the y-axis. Genome-wide significant SNPs with a significant heterogeneity Q (with a strong effect on one or some of the indicators that was not well explained through the common latent factor) are displayed in grey. The dashed line indicates the genome-wide significance threshold (*P* < 5e-8). *P*-values were computed using a two-sided Z-test. **a-b** Sample sizes for underlying GWAS summary statistics are reported in Supplementary Table 1. AF=Atrial Fibrillation; CAD=Coronary Artery Disease; HF=Heart Failure; MDD=Major depressive disorder; PAD=Peripheral Artery Disease. Source data

To further characterize the genetic overlap, we explicitly modeled the shared genetic liability between MDD and CVD as a higher-order latent factor using genomic SEM. We excluded atrial fibrillation because it deteriorated the model fit (comparative fit index (CFI) of 0.918 and standardized root mean squared residual (SRMR) of 0.072), so that the interpretation of the latent factor changed to representing ASCVD. The final model had an excellent fit (CFI of 0.999 and SRMR of 0.021). The loading of ASCVD on the MDD-ASCVD factor was *β* = 2.46 (95% CI [2.00, 2.91], *P* = 1.63 × 10^−26^). For model identification purposes, the loading of MDD was fixed to 1. Factor loadings for the ASCVDs on the ASCVD factor were *β* = 0.79 (95% CI [0.68, 0.90], *P* = 6.21 × 10^−45^) for stroke, *β* = 1.03 (95% CI [0.89, 1.17], *P* = 2.58 × 10^−46^) for peripheral artery disease, and *β* = 1.08 (95% CI [0.95, 1.21], *P* = 2.10 × 10^−59^) for heart failure, respectively (Fig. 3a; loadings are given standardized with respect to the genetic variance of the traits). For coronary artery disease, the factor loading was fixed to 1 for identification purposes. For comparison, we also fit a latent factor for the ASCVDs alone (without MDD), which showed similar fit and parameter estimates (CFI of >0.999 and SRMR of 0.013; Extended Data Fig. 3a). This shows that shared genetic liability to different ASCVDs as well as to ASCVDs and MDD (to a lesser extent) can be explained by a single underlying factor.

The GWAS on the latent MDD–ASCVD factor resulted in 205 independent genome-wide significant loci (Fig. 3b, independent at *R*^2^ < 0.1 and distance $\ge$ 250 kb; Supplementary Table 8). Almost three-quarters (74.6%) of the genome-wide statistically significant single-nucleotide polymorphisms (SNPs) showed a high *Q* heterogeneity, suggesting that their effects were more in line with an independent pathway than a common pathway model (Methods). Most of this heterogeneity was due to MDD, as the GWAS for latent ASCVD without MDD showed fewer genome-wide statistically significant SNPs with a high heterogeneity (30.6%; Extended Data Fig. 3b).

**Extended Data Fig. 4:**
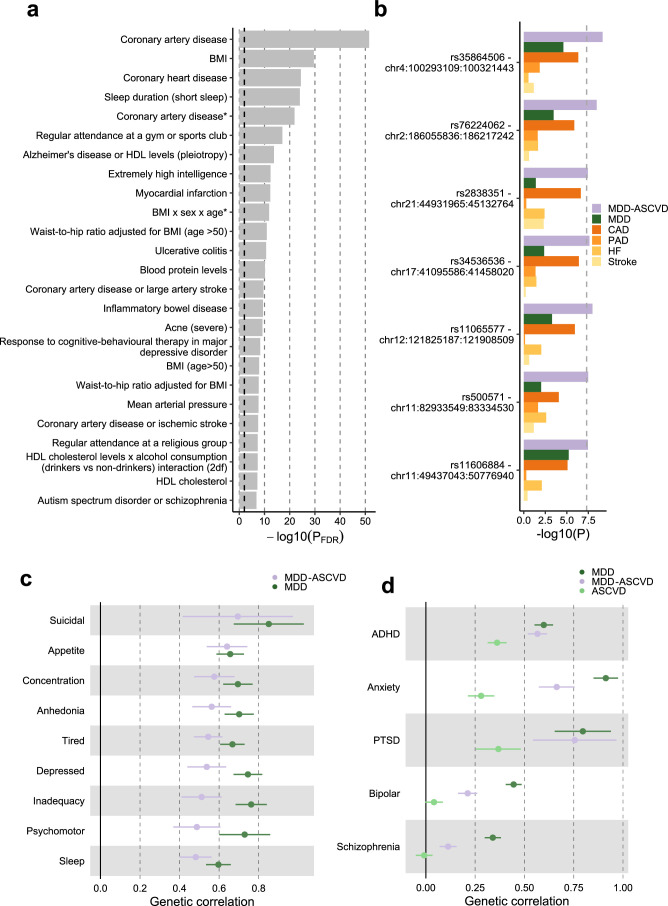
The genetic signature of MDD-ASCVD. **a**, Enrichment for the MDD-ASCVD GWAS SNPs in genome-wide significant SNPs for traits in the GWAS catalog computed using FUMA. The traits are as reported in the original study. Note that sleep duration here is a dichotomization of self-reported sleep. The dashed grey line indicates the significance threshold after FDR-adjustment. Adjustment for multiple testing was conducted over all traits in the GWAS catalog using the Benjamini-Hochberg procedure. *P*-values were computed using a one-sided hypergeometric test. **b**, The five SNPs that were significantly associated with MDD-ASCVD, but not with any of the constituent traits, with their *P*-value in the GWAS of the constituent traits. The dashed line indicates the genome-wide significance threshold (*P* < 5e-8). *P*-values from constituent traits are taken from the original GWAS, see Supplementary Table 1. *P*-values for MDD-ASCVD were computed using a two-sided Z-test **c**, Genetic correlation of MDD symptoms with MDD, and MDD-ASCVD. **d** Genetic correlation of MDD, MDD-ASCVD, ASCVD, with five mental disorders. **c-d** Points and error bars represent mean genetic correlation and 95% CIs. Results are computed using LD Score Regression analysis. **a-d** Sample sizes for underlying GWAS summary statistics are reported in Supplementary Table 1. ADHD = Attention Deficit and Hyperactivity Disorder; PTSD = Posttraumatic Stress Disorder. Source data

For the latent MDD–ASCVD factor, we filtered out variants that showed statistically significant heterogeneity and considered only variants where the latent MDD–ASCVD factor was the best model for the follow-up analyses. We retained 72 independent loci underlying the shared genetic liability (Fig. 3b and Supplementary Table 9). The top SNP after filtering was rs11670056 in the *ELL* gene, which encodes a part of the transcription elongation factor complex and has previously been associated with CVDs, blood traits, BMI and educational attainment (enrichment in associations with other traits for statistically significant SNPs are shown in Extended Data Fig. 4a). There were 19 top SNPs with significant expression quantitative trait loci (eQTL) for one or multiple genes (Fig. 3b and Supplementary Table 10). Besides *ELL*, multiple genes on chromosome 10 around *INA* and *CNNM2* were identified. *INA* and *CNNM2*, which encode a neurofilament and a protein involved in ion transportation, respectively, have previously been associated with psychiatric as well as cardiovascular traits.

We found seven novel loci in the latent MDD–ASCVD GWAS that were not among the risk loci in the MDD and ASCVD GWASs that constituted the latent factor (Extended Data Fig. 4b and Supplementary Table 11). The top SNPs in these loci have not been identified in any GWAS recorded in the GWAS catalog before, but four of them have shown suggestive associations (*P* < 0.05) with metabolic traits.

Using partitioned LDSC, we observed that the heritability of the latent MDD–ASCVD factor was enriched in genes with expression specific to endothelial tissues, which was also observed for latent ASCVD but not for MDD (Fig. 3c and Supplementary Table 12). To gain deeper insights into brain-specific mechanisms, we leveraged high-resolution human brain single-nucleus RNA sequencing data^27^ and identified four human brain cell types that exhibited enriched MDD–ASCVD heritability, including deep-layer corticothalamic and 6b cells, midbrain-derived inhibitory neurons, miscellaneous neurons and vascular cells (Fig. 3d and Supplementary Table 13). Notably, except for miscellaneous neurons, these cell types displayed no enrichment for either latent ASCVD or MDD, suggesting that the genetic variance for MDD–ASCVD comorbidity has a distinct functional signature.

To externally validate the MDD–ASCVD phenotype, we computed polygenic risk scores (PRS) based on the summary statistics for the latent MDD–ASCVD factor, as well as for MDD and latent ASCVD, and found them to predict ASCVD and MDD diagnoses in UK Biobank (UKB) (all *P* < 2 × 10^−13^; Fig. 3e and Supplementary Tables 14 and 15; note that source and target samples were overlapping and the *R*^2^ values should only be interpreted relative to one another). The PRS for latent ASCVD and the latent MDD–ASCVD factor explained similar amounts of variance in ASCVDs. This is in line with the MiXeR findings (Fig. 2b), suggesting that most causal variants for ASCVDs are shared with MDD. In contrast, as most causal variants for MDD are not shared with ASCVDs, the PRS for the latent MDD–ASCVD factor explained less than half as much variance in MDD as the MDD PRS.

Next, we assessed genetic correlations between the latent MDD–ASCVD factor and MDD symptoms. We found that poor appetite or overeating and suicidal thoughts are the symptoms most strongly correlated with MDD–ASCVD. In contrast, poor appetite or overeating is among the least genetically correlated symptoms to MDD (Extended Data Fig. 4c).

Finally, we estimated genetic correlation of attention deficit and hyperactivity disorder (ADHD), anxiety disorders, posttraumatic stress disorder (PTSD), bipolar disorder and schizophrenia, with MDD, latent MDD–ASCVD and latent ASCVD. We found that ADHD, anxiety disorder and PTSD were genetically correlated with latent ASCVD (Extended Data Fig. 4d). In addition, PTSD and ADHD showed similar genetic correlations for MDD and latent MDD–ASCVD, suggesting that variants that are shared between MDD and ASCVD might explain most of the genetic correlation between MDD and these disorders.

### Genetic overlap between MDD and risk factors

**Fig. 4:**
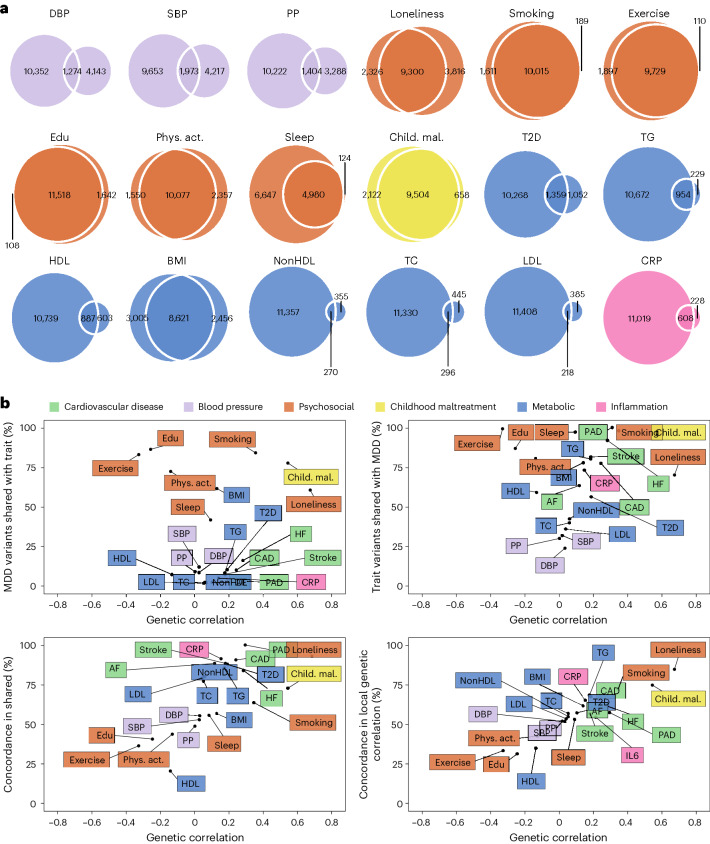
Local and causal-variant level genetic correlations between MDD and risk factors. **a**, Venn diagrams based on MiXeR results showing the number of causal variants (number of nonzero variants required to explain 90% of trait heritability) that are unique to MDD (left circle), the risk factor (nonoverlapping part of right circle) or shared between MDD and the risk factor (overlapping part of circles). **b**, Genome-wide genetic correlation estimated by LDSC (*r*_*g*_, *x* axis) against the percentage of MDD causal variants that are shared with the risk factor as estimated by MiXeR (top left), the percentage of risk factor causal variants that are shared with MDD (top right) and the percentage of shared variants that have concordant effect directions (bottom left). The bottom right plot shows the percentage of local genetic correlations from LAVA that have concordant effect directions on the *y* axis. Cardiovascular traits are also shown for comparison. For **a** and **b**, standard errors for MiXeR, LAVA and LDSC results are reported in Supplementary Tables 2–6. Sample sizes for GWAS summary statistics are reported in Supplementary Table 1. Note that IL6 was excluded from MiXeR results because it failed performance checks (Methods). Child. mal., childhood maltreatment; CRP, C-reactive protein; DBP, diastolic blood pressure; edu, educational attainment; HDL, high-density lipoprotein; LDL, low-density lipoprotein; nonHDL, non-high-density lipoprotein; phys. act., physical activity; PP, pulse pressure; psychosocial, psychosocial or lifestyle; SBP, systolic blood pressure; T2D, type II diabetes; TC, total cholesterol; TG, triglycerides. Source data

**Extended Data Fig. 5:**
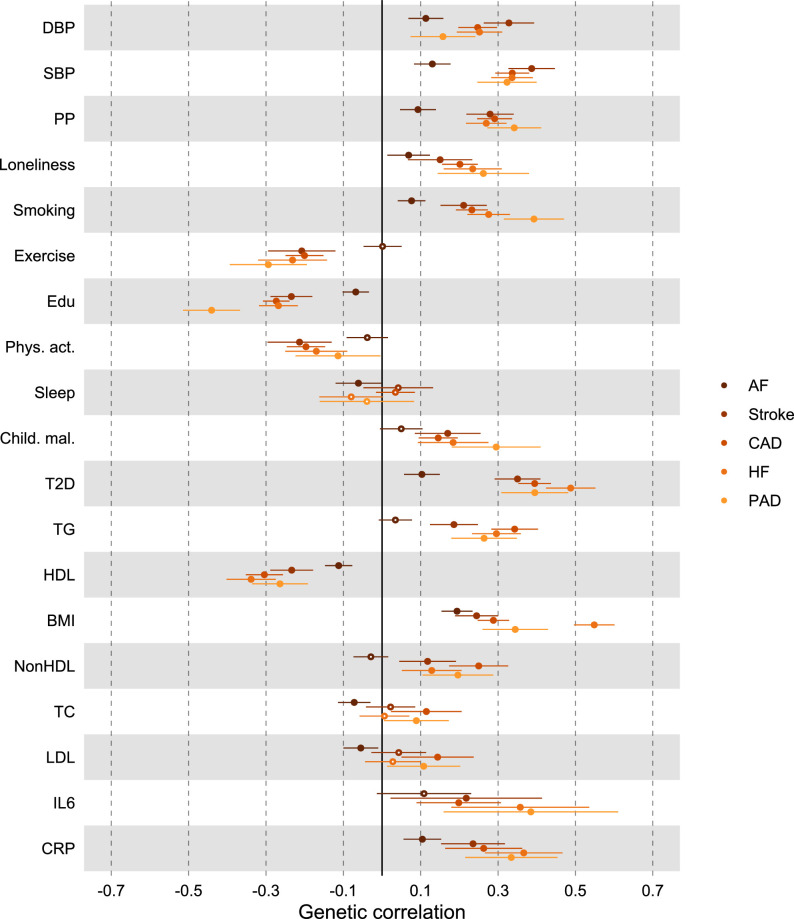
Genetic correlation between CVDs and risk factors. Results are based on LD Score Regression analysis. Points and error bars represent mean genetic correlation and 95% CIs. Sample sizes for underlying GWAS summary statistics are reported in Supplementary Table 1. Open dots indicate a non-significant genetic correlation. AF=Atrial Fibrillation; CAD=Coronary Artery Disease; Child. Mal.=Childhood Maltreatment; CRP = C-Reactive Protein; DBP=Diastolic Blood Pressure; Edu=Educational attainment; HDL=High-Density Lipoprotein; HF=Heart Failure; IL6=Interleukin-6; LDL=Low-Density Lipoprotein; MDD=Major Depressive Disorder; NonHDL=Non-High-Density Lipoprotein; PAD=Peripheral Artery Disease; Phys. Act.=Physical activity; PP=Pulse Pressure; Psychosocial=Psychosocial/lifestyle; SBP=Systolic Blood Pressure; T2D=Type II Diabetes; TC=Total Cholesterol; TG=Triglycerides. Source data

**Extended Data Fig. 6:**
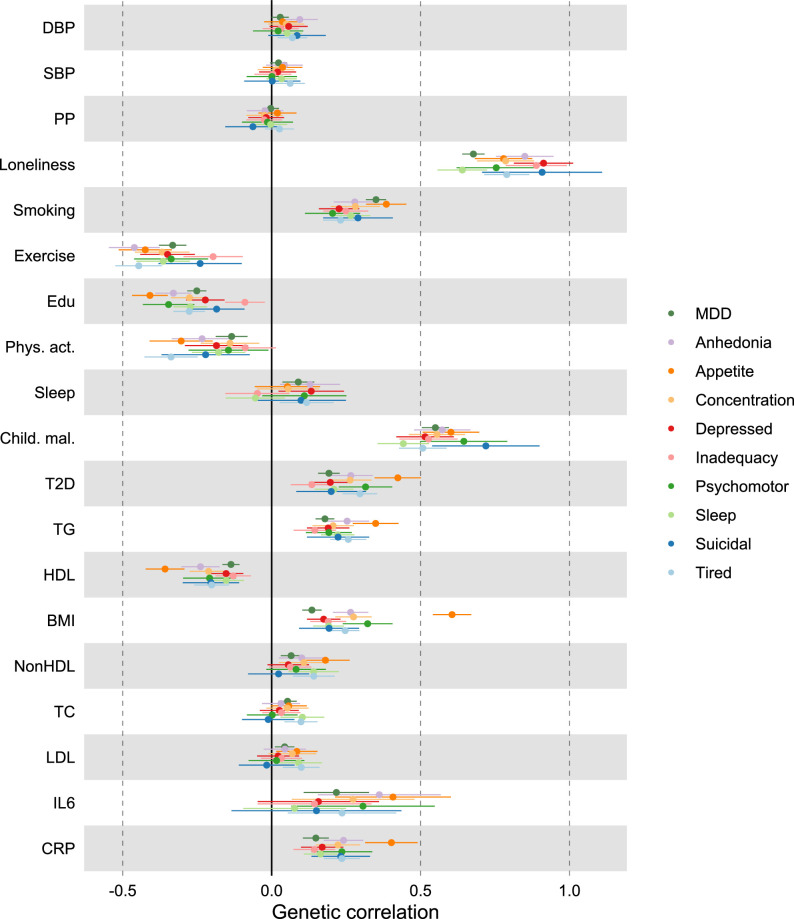
Genetic correlation between MDD traits and risk factors. Results are based on LD Score Regression analysis. Points and error bars represent mean genetic correlation and 95% CIs. Sample sizes for underlying GWAS summary statistics are reported in Supplementary Table 1. Open dots indicate a non-significant genetic correlation. AF=Atrial Fibrillation; CAD=Coronary Artery Disease; Child. Mal.=Childhood Maltreatment; CRP = C-Reactive Protein; DBP=Diastolic Blood Pressure; Edu=Educational attainment; HDL=High-Density Lipoprotein; HF=Heart Failure; IL6=Interleukin-6; LDL=Low-Density Lipoprotein; MDD=Major Depressive Disorder; NonHDL=Non-High-Density Lipoprotein; PAD=Peripheral Artery Disease; Phys. Act.=Physical activity; PP=Pulse Pressure; Psychosocial=Psychosocial/lifestyle; SBP=Systolic Blood Pressure; T2D=Type II Diabetes; TC=Total Cholesterol; TG=Triglycerides. Source data

Next, we aimed to identify risk factors linking MDD and CVD. First, we assessed genetic correlations between MDD and risk factors. We observed strong to moderate genetic correlations of MDD with psychosocial or lifestyle factors, such as loneliness (*r*_*g*_ = 0.68, 95% CI [0.64, 0.72]), childhood maltreatment (*r*_*g*_ = 0.55, 95% CI [0.50, 0.60]) and exercise (*r*_*g*_ = −0.33, 95% CI [−0.38, −0.29]) (Fig. 4b and Extended Data Fig. 6). Among metabolic factors, MDD showed the strongest genetic correlation with type II diabetes (*r*_*g*_ = 0.19, 95% CI [0.16, 0.23]) as well as levels of high-density lipoprotein cholesterol (*r*_*g*_ = −0.14, 95% CI [−0.17, −0.11]) and triglycerides (*r*_*g*_ = 0.18, 95% CI [0.15, 0.21]). We observed genetic correlation of MDD with levels of the inflammatory markers IL6 (*r*_*g*_ = 0.22, 95% CI [0.11, 0.33]) and C-reactive protein (*r*_*g*_ = 0.15, 95% CI [0.10, 0.19]). We did not observe genetic correlations of MDD with blood pressure traits. As a comparison, for CVDs, the largest genetic correlations were found between heart failure and BMI (*r*_*g*_ = 0.55, 95% CI [0.50, 0.60]) and type II diabetes (*r*_*g*_ = 0.49, 95% CI [0.42, 0.55]) (Extended Data Fig. 5 and Supplementary Table 2). The results for MDD symptoms largely followed the pattern of MDD diagnosis, although poor appetite or overeating showed stronger genetic correlations with metabolic factors than did MDD diagnosis (Extended Data Fig. 6 and Supplementary Table 3).

**Extended Data Fig. 7:**
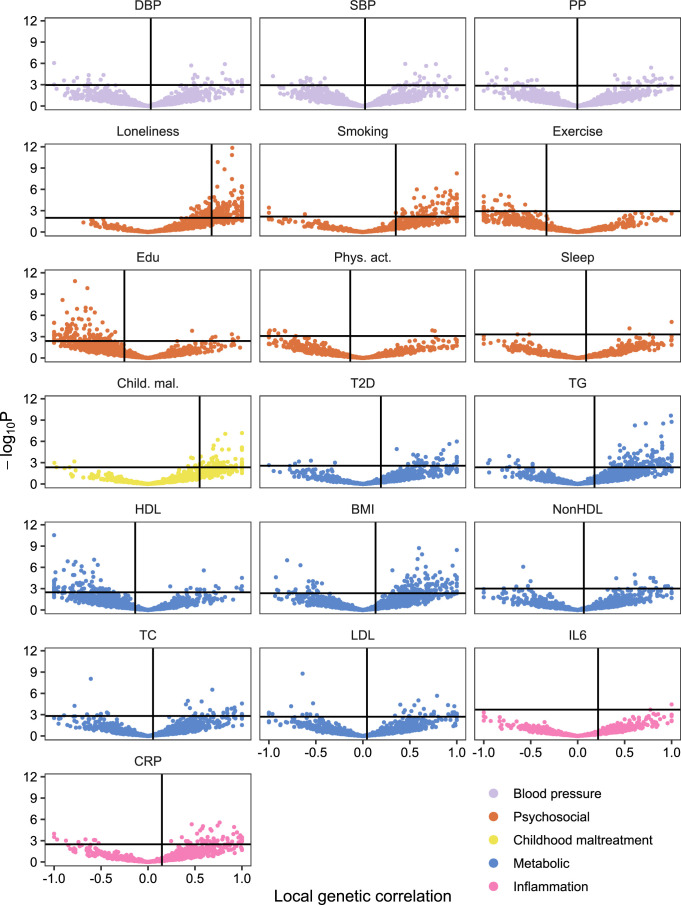
Local genetic correlations between MDD and risk factors. Volcano plots based on LAVA results. Local genetic correlation between MDD and each of the risk factors (x-axis) and the corresponding -log10 transformed *P*-value (y-axis). Empirical P-values were obtained via a permutation procedure with partial integration, evaluating the two-sided hypothesis of no association using the estimated parameters as test statistics. Correlations were estimated in the loci that showed marginally significant local heritability in 2,495 considered genomic regions. Loci exceeding the horizontal line are significant at ${P}_{{\rm{FDR}}}$<.05. Multiple testing was adjusted individually for each trait over considered loci using the Benjamini-Hochberg procedure. Sample sizes for underlying GWAS summary statistics are reported in Supplementary Table 1. Child. Mal.=Childhood Maltreatment; CRP = C-Reactive Protein; DBP=Diastolic Blood Pressure; Edu=Educational attainment; HDL=High-Density Lipoprotein; IL6=Interleukin-6; LDL=Low-Density Lipoprotein; MDD=Major Depressive Disorder; NonHDL=Non-High-Density Lipoprotein; Phys. Act.=Physical activity; PP=Pulse Pressure; Psychosocial=Psychosocial/lifestyle; SBP=Systolic Blood Pressure; T2D=Type II Diabetes; TC=Total Cholesterol; TG=Triglycerides. Source data

Causal-variant and local genetic correlation analysis revealed several patterns of genetic overlap between MDD and risk factors. We found that psychosocial or lifestyle factors, childhood maltreatment and BMI showed similar levels of polygenicity to MDD (Extended Data Fig. 2b and Supplementary Tables 5 and 7). In addition, they exhibited a large degree of shared variants and many local genetic correlations with MDD (Fig. 4a, Extended Data Fig. 7 and Supplementary Tables 4 and 6). Out of these factors, loneliness and childhood maltreatment also showed high levels of effect direction concordance with MDD, both for risk variants and local genetic correlations (Fig. 4b and Supplementary Tables 4 and 6). Combined with high polygenicity, such concordance translates to large genome-wide genetic correlations. In contrast, educational attainment, smoking, exercise, physical activity, BMI and sleep duration had similar levels of polygenicity and a large degree of polygenic overlap with MDD, but low effect direction concordance, suggesting that genome-wide genetic correlations underestimate the genetic overlap with MDD for these traits.

Genetic factors for blood pressure traits showed unique patterns of genetic overlap with MDD. They were polygenic (>5,000 causal variants; Extended Data Fig. 2b) but did not overlap strongly with genetic risk factors for MDD (<0.30 of risk variants overlapping; Fig. 4a). Moreover, risk variants that did overlap showed low degree of effect direction concordance (48–57% of shared variants in the same direction; Fig. 4b). We observed 97 significant local genetic correlations with MDD for the three blood pressure traits, 60% of which were positive. These findings suggest that MDD and blood pressure share variants that exhibit both positive and negative correlations, which are canceled out in the genome-wide estimate.

Type II diabetes, lipid traits and C-reactive protein showed low polygenicity (<2,500 causal risk variants). Type II diabetes, triglyceride levels and C-reactive protein levels shared most of their risk variants with MDD, and these variants showed high degrees of concordance (>85% of shared variants in the same direction). High-density lipoprotein cholesterol shared most of its risk variants with MDD, in consistently opposite directions.

### Risk factors underlying genetic overlap between MDD and CVD

**Fig. 5:**
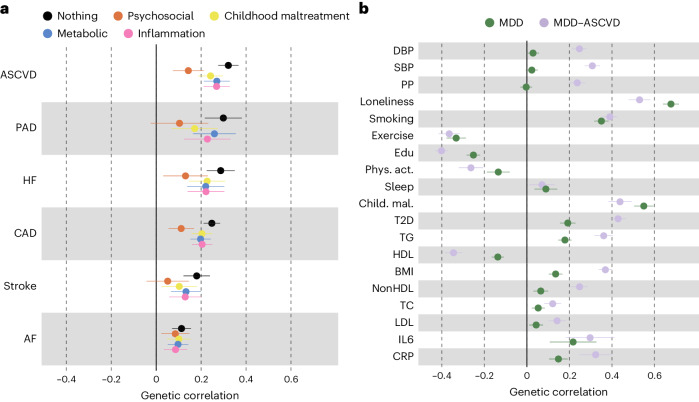
Genetic correlation between MDD and CVD explained by risk factors. **a**, The genetic correlation between MDD and CVD before and after adjustment for groups of risk factors (color coded). **b**, A comparison of genetic correlation between MDD (dark green) and the latent MDD–ASCVD factor (lilac) and individual risk factors. In **a** and **b** the points and error bars represent mean genetic correlation and 95% CIs. The sample sizes for GWAS summary statistics are reported in Supplementary Table 1. Source data

**Extended Data Fig. 8:**
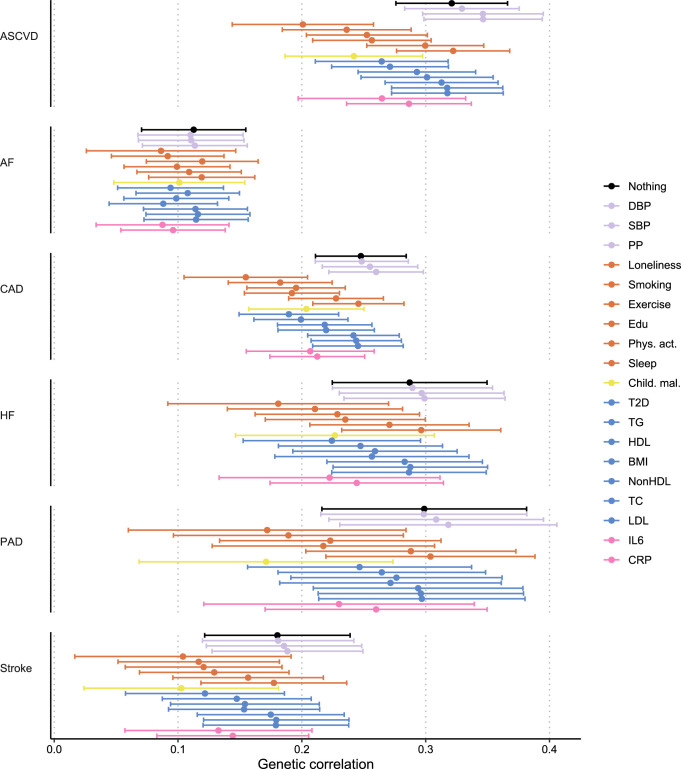
Genetic correlation between MDD and CVD adjusting for individual risk factors. Results from Genomic SEM. Points and error bars represent mean genetic correlation and 95% confidence intervals. Reference estimates of the association without any adjustment are printed in black. Sample sizes for underlying GWAS summary statistics are reported in Supplementary Table 1. AF=Atrial Fibrillation; CAD=Coronary Artery Disease; Child. Mal.=Childhood Maltreatment; CRP=C-Reactive Protein; DBP=Diastolic Blood Pressure; Edu=Educational attainment; HDL=High-Density Lipoprotein; HF=Heart Failure; IL6=Interleukin-6; LDL=Low-Density Lipoprotein; MDD=Major Depressive Disorder; NonHDL=Non-High-Density Lipoprotein; PAD=Peripheral Artery Disease; Phys. Act.=Physical activity; PP=Pulse Pressure; Psychosocial=Psychosocial/lifestyle; SBP=Systolic Blood Pressure; T2D=Type II Diabetes; TC=Total Cholesterol; TG=Triglycerides. Source data

To assess risk factors explaining the genetic overlap between MDD and CVD, we estimated genetic correlations adjusted for risk factors (individually or as a group) using genomic SEM (Fig. 5a, Extended Data Fig. 8 and Supplementary Table 16). The largest reduction in point estimate was observed for the genetic correlation between MDD and peripheral artery disease after adjustment for the group of psychosocial or lifestyle factors. Similarly, the genetic correlation of MDD with coronary artery disease and the latent ASCVD factor were attenuated after adjusting for psychosocial or lifestyle factors. The reduction was mainly driven by loneliness (Extended Data Fig. 8). Genetic correlations of MDD with peripheral artery disease and stroke were no longer statistically significant after adjusting for psychosocial or lifestyle factors. We also observed some attenuation in the genetic correlation between MDD and CVDs after adjusting for childhood maltreatment, metabolic factors or inflammatory markers, although confidence intervals (CIs) overlapped.

Next, we specified the risk factors as mediators instead of covariates in the genomic SEM model and compared the path estimates from MDD with CVD. Observing attenuation in the association between MDD and CVD when a risk factor is modeled as a mediator rather than a covariate supports the interpretation that the risk factor mediates some of the link between MDD and CVD. For all psychosocial or lifestyle factors together, the inflammation traits, and for type II diabetes, the 95% CIs did not overlap between the mediator and covariate models, suggesting that these risk factors are mediating part of the link between MDD and CVD (Supplementary Table 17).

Finally, we estimated genetic correlations between the risk factors and the latent MDD–ASCVD factor (Fig. 5b) and found that the latent MDD–ASCVD factor was substantially more genetically correlated with blood pressure traits, C-reactive protein levels and metabolic factors than MDD only, suggesting that these factors characterize the genetic liability to MDD–ASCVD rather than to MDD alone.

### Causal pathways linking MDD and CVD

**Fig. 6:**
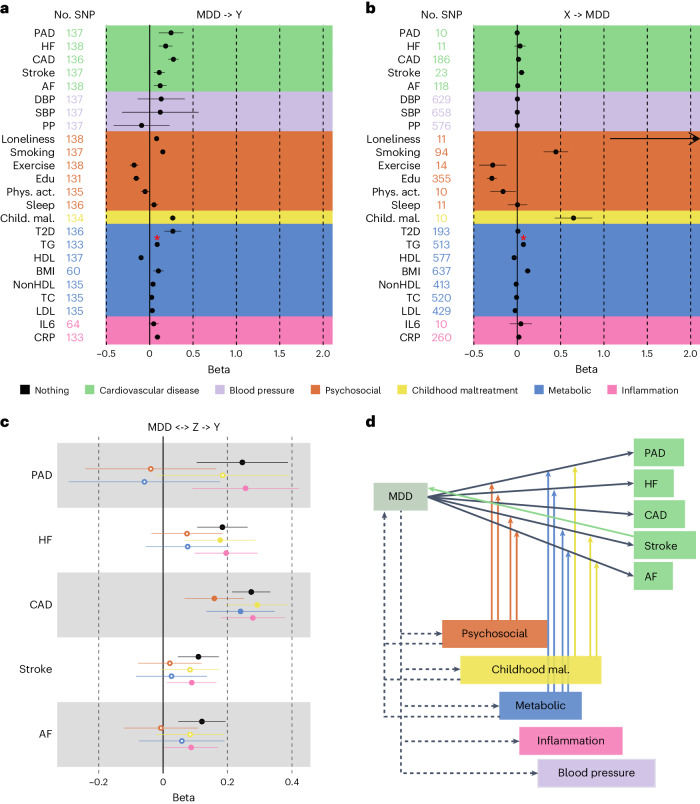
Support for causal effects between MDD, CVD and shared risk factors. **a**, The effect of genetic instruments for MDD (exposure) on CVD and risk factors (outcomes). **b**, The effect of genetic instruments for CVDs and risk factors on MDD. The arrow indicates that the CI for the effect of loneliness on MDD has been cut to improve readability. **c**, The effects of genetic instruments for MDD on CVD while adjusting for groups of risk factors in multivariable MR. **d**, A schematic overview of levels of evidence for causal effects, with solid lines indicating convincing evidence (consistent across sensitivity analyses) for such effects and dashed lines indicating evidence for some of the relationships tested within the trait categories. The arrows from the risk factors to the association between MDD and the CVDs indicate that the combined risk factors attenuated the association so that it was no longer statistically significant. In **a**–**c**, the IVW estimate is shown. The results from the sensitivity analyses are reported in Supplementary Table 18. In **a**–**d**, the points and error bars represent mean effect size (regression coefficient) and 95% CIs. The sample sizes for GWAS summary statistics are reported in Supplementary Table 1. The term beta refers to the log odds ratio. The asterisk (*) indicates that the observed statistically significant association suffered from pleiotropy; possible causal effect should not be interpreted. Source data

We investigated putative causal relationships between MDD and CVD using two-sample MR. Instruments were Steiger filtered, that is, SNPs explaining statistically significantly more variance in the outcome than the exposure were excluded. The results provide support for a causal effect of MDD liability on all CVDs, with the strongest effects observed for coronary and peripheral artery disease (Fig. 6a). We found no statistically significant pleiotropy and the results were consistent across weighted median, mode and Egger sensitivity analyses, providing support for the inverse variance weighted (IVW) estimates (Supplementary Table 18).

For risk factors, we observed that increased liability to MDD was associated with increased loneliness, smoking, risk of type II diabetes and levels of C-reactive protein (Fig. 6a). These results were again consistent across sensitivity analyses (Supplementary Table 18).

We also investigated potential causal effects in the other direction with genetic instruments to CVDs and risk factors as exposures and MDD as outcome (Fig. 6b). We provide evidence for a statistically significant causal effect of stroke, loneliness, smoking, exercise, educational attainment, childhood maltreatment, levels of high-density lipoprotein cholesterol and BMI on MDD risk (Fig. 6b), which was robust across sensitivity analyses (Supplementary Table 18). No robust effects were observed for other CVDs, blood pressure, other metabolic traits or inflammatory markers.

**Extended Data Fig. 9:**
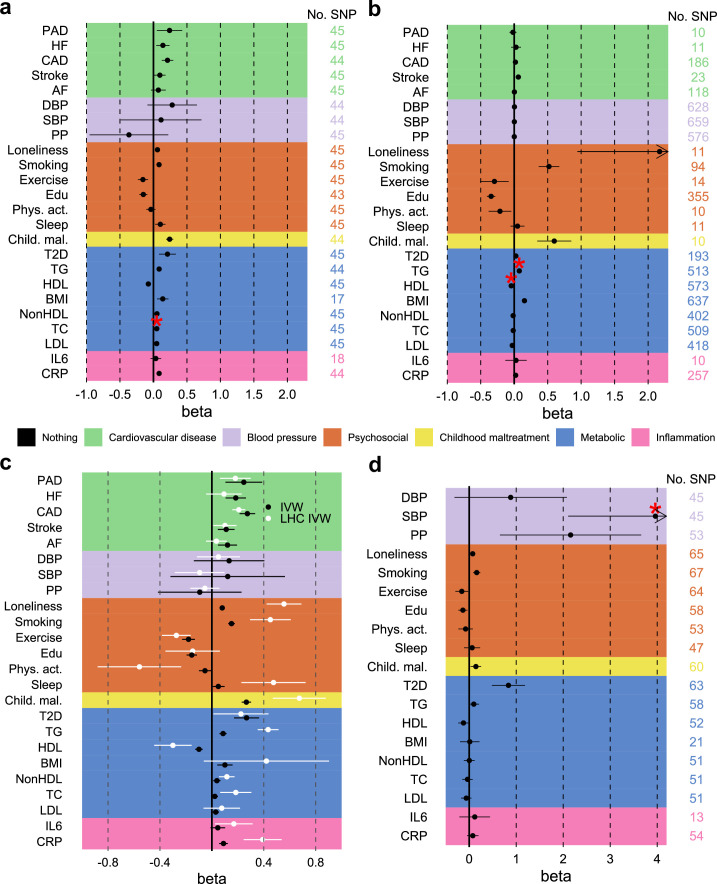
Sensitivity analyses providing additional support for causal effects between MDD, CVD, and shared risk factors. **a**, Effect of genetic instruments for MDD (exposure) on CVD and risk factors (outcomes) after excluding the UKB sample from the MDD GWAS (which was responsible for most of the sample overlap in the exposure and outcome GWAS summary statistics). **b**, Effect of genetic instruments for CVD and risk factors on MDD after excluding the UKB sample from MDD GWAS. **c**, Results from LHC MR, that corrects for heritable confounders and sample overlap, with the IVW MR estimate given as reference, for the effect of MDD on CVD and risk factors. **d** Effect of genetic instruments for MDD-ASCVD on outcomes and risk factors. **a-d** Significantly pleiotropic estimates are indicated with a red asterisk. IVW estimate shown. Results from sensitivity analyses are reported in Supplementary Table 20. Points and error bars represent mean effect size (regression coefficient) and 95% CIs. Sample sizes for GWAS summary statistics are reported in Supplementary Table 1. The term beta refers to the log odds ratio. AF=Atrial Fibrillation; CAD=Coronary Artery Disease; Child. Mal.=Childhood Maltreatment; CRP = C-Reactive Protein; DBP=Diastolic Blood Pressure; Edu=Educational attainment; HDL=High-Density Lipoprotein; HF=Heart Failure; IL6=Interleukin-6; LDL=Low-Density Lipoprotein; MDD=Major Depressive Disorder; NonHDL=Non-High-Density Lipoprotein; PAD=Peripheral Artery Disease; Phys. Act.=Physical activity; PP=Pulse Pressure; Psychosocial=Psychosocial/lifestyle; SBP=Systolic Blood Pressure; T2D=Type II Diabetes; TC=Total Cholesterol; TG=Triglycerides. Source data

When using genetic instruments for the latent MDD–ASCVD factor to predict risk factors, statistically significant effects were observed for pulse pressure and type II diabetes (Extended Data Fig. 9d). We found statistically significant pleiotropy for systolic blood pressure.

**Extended Data Fig. 10:**
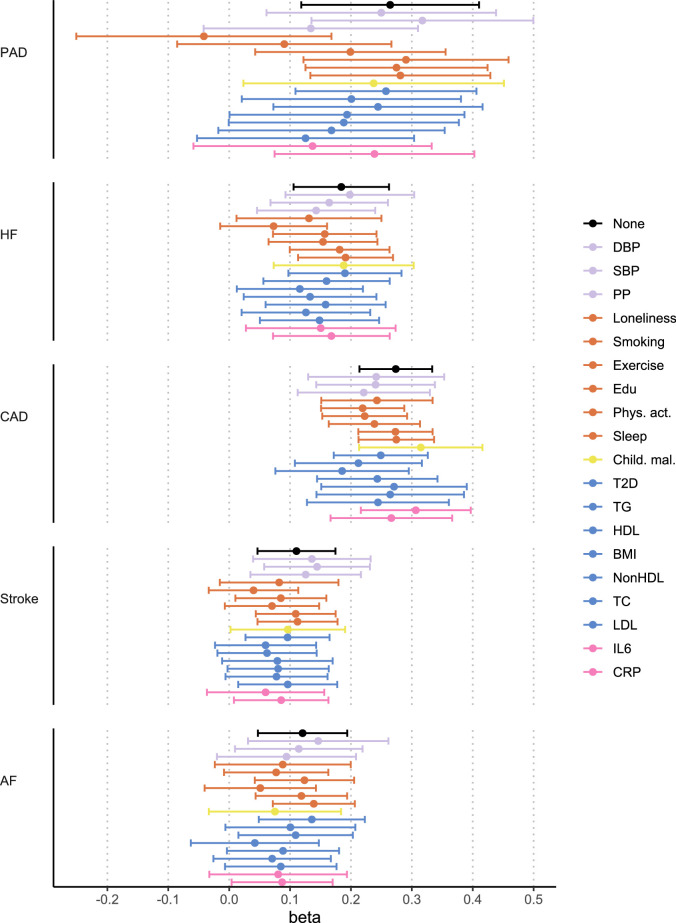
Support for causal effect of MDD on CVDs when adjusting for individual risk factors. Results from multivariable MR. Points and error bars represent mean effect size (regression coefficient) and 95% CIs. IVW estimate shown. Reference estimates of the association without any adjustment are printed in black. Sample sizes for GWAS summary statistics are reported in Supplementary Table 1. The term beta refers to the log odds ratio. AF=Atrial Fibrillation; CAD=Coronary Artery Disease; Child. Mal.=Childhood Maltreatment; CRP=C-Reactive Protein; DBP=Diastolic Blood Pressure; Edu=Educational attainment; HDL=High-Density Lipoprotein; HF=Heart Failure; IL6=Interleukin-6; LDL=Low-Density Lipoprotein; MDD=Major Depressive Disorder; NonHDL=Non-High-Density Lipoprotein; PAD=Peripheral Artery Disease; Phys. Act.=Physical activity; PP=Pulse Pressure; Psychosocial=Psychosocial/lifestyle; SBP=Systolic Blood Pressure; T2D=Type II Diabetes; TC=Total Cholesterol; TG=Triglycerides. Source data

We extended univariable results with multivariable MR to assess whether there was support for causal effects of MDD on CVD explained by the risk factors. We included only risk factors that were associated with MDD in the univariable MR analysis. The effects of MDD on CVDs were attenuated after adjusting for the psychosocial or lifestyle risk factors, driven by loneliness and smoking, and metabolic risk factors, although CIs were wide (Fig. 6c, Extended Data Fig. 10 and Supplementary Table 19). The effect of MDD liability on peripheral artery disease, heart failure, stroke and atrial fibrillation risk was no longer statistically significant after adjusting for the groups of metabolic or psychosocial or lifestyle factors (Fig. 6d).

To investigate possible bias due to sample overlap, we repeated the analyses using MDD summary statistics based on GWAS excluding the UKB sample (the main source of overlap). We observed small differences in the estimates, but the interpretation remained the same for all results (Extended Data Fig. 9a,b and Supplementary Table 20). Furthermore, we repeated the analyses using latent heritable confounder (LHC) MR, which is robust to sample overlap^47^. The results pattern was similar, although the point estimates were slightly attenuated for CVD risk (for example, no longer statistically significant for heart failure and atrial fibrillation) and became stronger for most other traits (Extended Data Fig. 9c). We conclude that although sample overlap impacted the point estimates, the interpretation of results remained similar.

## Discussion

Here, we showed that genetic risk factors for CVD overlap strongly with MDD. We modeled the shared genetic liability between MDD and ASCVD as a latent factor and showed that, distinct from MDD alone, it is associated with gene expression specific to thalamic and vascular cell types in the brain and is genetically correlated with immunometabolic factors and blood pressure. Further, we showed that the association between MDD and CVD is partly explained by modifiable risk factors and provide evidence that it is probably causal in nature.

In line with previous results^22,48–50^ we found moderate genome-wide genetic correlations between MDD and CVDs. Analysis on the level of shared risk variants showed that MDD was substantially more polygenic than the CVD traits and that most risk variants for CVD were in fact shared with MDD and had concordant effect directions. In addition, we found many positive local genetic correlations between MDD and CVD, especially for coronary artery disease, although that might reflect the fact that the coronary artery disease GWASs have larger sample size than the GWASs of the other CVDs. Interestingly, we found that the *HLA* region was a hotspot for local genetic correlation between MDD and CVDs. These findings suggest that genetic overlap between MDD and CVD is underestimated in genome-wide correlation analyses.

We modeled the shared liability between MDD and ASCVD as a latent MDD–ASCVD factor and performed a GWAS on the factor. Atrial fibrillation was excluded because it deteriorated the fit of the latent factor, which was in line with results that showed that atrial fibrillation was substantially less genetically correlated with the ASCVDs than they were with each other. Combined, these results suggest that atrial fibrillation and ASCVDs have partly distinct sources of genetic variation. We identified many loci associated with MDD–ASCVD, some of which were uniquely associated with the shared liability and not with the constituent traits. We found that heritability for the MDD–ASCVD latent factor was enriched for genes specifically expressed in vascular braincells, deep-layer corticothalamic 6b (projecting to the thalamus) and midbrain-derived inhibitory neurons (predominantly located in the thalamus). This cell-type enrichment signature was not found for MDD or ASCVD alone, suggesting that a distinct mechanism involving thalamic circuits might underlie the shared liability to MDD–ASCVD. Altered thalamic function has indeed been implicated previously in CVD^51–53^ and MDD^54,55^, and white matter integrity in thalamic radiations show associations with aortic area and myocardial wall thickness, suggesting that it has a role in the ‘heart–brain’ connection^56^.

MDD showed a high degree of genetic overlap with risk factors. We showed that MDD was substantially more polygenic than blood pressure, lipid and inflammatory traits. In contrast, psychosocial or lifestyle traits were equally polygenic to MDD and showed a large degree of overlap with MDD. Interestingly, the local and variant-level analysis indicated that blood pressure traits shared a substantial number of risk variants with MDD (in line with a previous report^49^), which was masked at the genome-wide level due to their opposing effect directions. Similarly, BMI and lipid traits showed discordant directions to MDD in effects of overlapping risk variants and local genetic correlations. Finally, C-reactive protein shared most of its risk variants with MDD, predominantly in the positive direction, indicating a genetic relationship between C-reactive protein and MDD that was masked in the genome-wide genetic correlation estimate. Overall, these findings refine our understanding of the polygenic overlap between MDD and risk factors shared between MDD and CVD, and indicate that it is stronger and more complex than has previously been reported.

We estimated genetic correlations between MDD and CVD adjusting for risk factors and found that psychosocial or lifestyle factors explain a substantial part of the genetic correlation between MDD and CVD and highlight loneliness as an important factor in the relationship between MDD and CVD. In addition, we found tentative support for a mediating role of C-reactive protein and IL6 levels in the association between MDD and ASCVD.

We found that, compared with MDD, the latent MDD–ASCVD factor was characterized by genetic correlations with immunometabolic factors and blood pressure, suggesting that the shared genetic liability to MDD and ASCVD is associated with an immunometabolic subtype of depression. The existence of an immunometabolic subtype of depression has been proposed previously, based on a long line of evidence of oxidative stress and neuroendocrine and inflammatory dysregulation in MDD that are preferentially associated with atypical symptoms of MDD (for example, weight gain and oversleeping)^57^. Indeed, we find that poor appetite or overeating is the MDD symptom with the consistently greatest genetic correlations to the CVDs, and it is among the most genetically correlated symptoms with MDD–ASCVD, while it is among the least genetically correlated symptoms to MDD, although CIs were too wide to be conclusive. We did not find large genetic correlations between sleep duration measured using an accelerometer over 1 week and the MDD–ASCVD factor. However, statistically significant variants for the MDD–ASCVD factor were strongly enriched in statistically significant variants for self-reported short sleep duration (<6 h per night)^58^ suggesting that short sleep duration might be more related to MDD–ASCVD comorbidity than overall sleep duration.

Mental disorders that are highly comorbid with MDD, such as psychotic disorders, anxiety disorders and PTSD, have also been shown to be associated with CVD^59^. We find that ADHD, anxiety disorders and PTSD show genetic correlations with ASCVD and MDD–ASCVD. For ADHD and PTSD, the genetic correlation was similar between MDD and MDD–ASCVD. Future work should estimate latent factors representing shared and distinct sources of genetic covariance among MDD, ADHD, anxiety disorders and PTSD and investigate how those factors relate to ASCVD.

We found robust support for the likelihood of causal effects of MDD on CVD. Previous two-sample MR studies have observed associations between genetic liability to MDD and risk of coronary artery disease but results for heart failure and stroke have been inconsistent^21,22,48,60^. Using more recent GWAS data, we confirmed an effect of genetic liability to MDD on coronary artery disease and found robust associations for stroke, heart failure and peripheral artery disease.

Except for stroke, we found limited evidence for a causal effect of CVDs on MDD, which is contrary to literature suggesting that such effects exist^23^. The MDD sample is mainly based on large volunteer-based studies that might select against individuals with CVD. Indeed, participants in the UKB study are healthier than the general population^61^. In addition, interpretation of the MR estimate in this case is complicated by the fact that CVD is a time-varying exposure with late age of onset^62^. However, we do find that genetic instruments capture the well-established association between stroke and subsequent MDD^63^. Therefore, the findings in our study offer some indication that the association between CVDs and subsequent MDD might have been overestimated in previous studies, possibly due to reverse causation, surveillance bias or unmeasured confounding.

We observed effects of genetic liability to MDD on most of the risk factors. We did not observe associations between genetic liability to MDD and blood pressure traits, although the presence of correlated and anticorrelated genetic components complicates interpretation. Indeed, using genetic instruments for the latent MDD–ASCVD factor, we did observe strong associations for pulse pressure. Previous MR studies of the association of C-reactive protein and IL6 levels with MDD risk have shown inconsistent results^64,65^. We did not find an effect of genetic instruments for inflammatory markers on MDD. However, we did find associations between genetic liability to MDD and increased C-reactive protein levels, lipid levels and type II diabetes, offering evidence that MDD might lead to long-term dysregulated immunometabolic pathways^57^. Likewise, in line with previous evidence^66^, we find support for a causal effect of liability to MDD on smoking, which, in turn, can lead to inflammation.

On multivariable MR analysis, we observed that only the association between genetic liability to MDD and coronary artery disease remained statistically significant after adjusting for the psychosocial or lifestyle or metabolic covariates. We found that adjusting for smoking status attenuates the association between genetic liability to MDD and peripheral artery disease, for which smoking is a strong risk factor^67^. Interestingly, we find that loneliness is an equally important factor explaining the relationship between MDD and peripheral artery disease. This emphasizes the need for interventions and preventive policies for reducing loneliness in the population, which has further increased in prevalence during the coronavirus disease 2019 pandemic and has been described as a pandemic itself^68,69^.

For most CVDs, no risk factor group could fully explain the genetic association between MDD and the CVD, which suggests the existence of additional mechanisms that are not captured by the genetic data used in the study. For instance, GWAS measure lifetime genetic risk and cannot capture dynamic processes of cumulative and interactive risk. Future studies should validate our findings using longitudinal data. Follow-up studies could also investigate the clinical usefulness of PRS for the shared liability to MDD–ASCVD and evaluate their ability to identify individuals at risk for immunometabolic depression, as we were unable to investigate this due to sample overlap in the present study. Another limitation is that the MiXeR model was not able to accurately estimate the genetic overlap between MDD and IL6. The likely reason the MiXeR model failed in this case is the markedly different genetic architectures of the two traits, with MDD being highly polygenic and IL6 being the least polygenic of the traits in the study, combined with the low sample size of the IL6 GWAS. Finally, to assess generalizability, these findings should be replicated with data from different ancestry sources. The lack of large genetic datasets from non-European populations is a crucial limitation that is widely acknowledged and yet difficult to circumvent. Observational studies have shown that MDD and CVDs could demonstrate different associations depending on ancestry^70,71^, and more research is needed to understand such differences^22,48–50^.

Our findings suggest that the shared genetic liability to MDD and ASCVD has a distinct genomic signature compared with MDD or ASCVD separately. Moreover, the shared genetic liability shows stronger genetic correlations with immunometabolic risk factors than MDD alone, in line with the idea of an inflammatory^72^ or immunometabolic^57^ subtype of MDD especially associated with ASCVDs, highlighting the role of inflammation in MDD–ASCVD comorbidity. Indeed, we found that the HLA region is a hotspot of local genetic correlation between MDD and the CVD traits, that genetic liability to MDD is associated with C-reactive protein levels and tentative support that inflammatory markers mediate some of the link between MDD and ASCVD. We highlight loneliness and smoking as important targets for intervention to reduce the risk of MDD and CVD, as well as CVD in individuals with MDD. Building on this work, tools can be developed to identify individuals at risk for developing immunometabolic depression (for example, using blood tests of high-density lipoprotein and C-reactive protein levels) and target them for cholesterol-lowering or anti-inflammatory medical interventions.

## Methods

### Data sources

All data sources were summary statistics from the largest and most recent GWAS so far (Supplementary Table 1). The MDD symptoms GWAS have not been published, although they have been used in other publications^64^. The symptom GWASs were based on Patient Health Questionnaire (PHQ-9) items measured in the UKB, that captures most, but not all, core symptoms of MDD and are available online^73^. The disease trait GWASs were mostly based on electronic health record diagnoses. The physical activity and sleep duration traits were measured in the UKB using an accelerometer over a 1-week period^36^.

The summary statistics were processed using the cleansumstats pipeline (https://github.com/BioPsyk/cleansumstats). SNPs were aligned and harmonized against dbSNP reference data. Analyses were conducted on the Tjenester for Sensitive Data cluster, maintained by the University of Oslo, using singularity containers. Containers are applications packaged with environmental dependencies to facilitate standardization of analyses across different sites, ensuring correct software versions and parameters^74^. All GWASs were ethically approved and were conducted in compliance with ethical guidelines. Ethics approval for the UKB study was given by the North West Centre for Research Ethics Committee (11/NW/0382). The work described here was approved by UKB under application number 22224.

### Genetic overlap

#### Genetic correlation

We estimated genetic overlap on the genome-wide, polygenic and local levels. For the genome-wide level, we estimated genetic correlations using LD score regression (LDSC^45^). We excluded the HLA region from the analysis because its complex LD structure can bias both heritability and genetic correlation results^75^. Note that LDSC performs well in the presence of sample overlap.

#### Local genetic correlation

We used LAVA^25^ to assess genetic correlation in regions of the genome. We assessed local genetic correlation in 2,495 genomic regions that cover the autosomes and have been defined to minimize LD between the regions while simultaneously keeping the regions approximately equal in size. These regions are provided with the LAVA software package. We only considered local genetic correlation in loci where both traits showed marginally significant heritability (*P* < 0.05). For these loci, we adjusted local genetic correlation *P* values for multiple testing using the Benjamini–Hochberg method. This adjustment was done separately for all pairs of traits considered. To match the results from LDSC and MiXeR (see below), we excluded the *HLA* region from the main analyses. For pairwise local genetic correlations between MDD and the CVD traits, we performed an additional analysis in the HLA region.

#### Genetic overlap

To investigate genetic overlap beyond genetic correlation, we used MiXeR v1.3 (ref. ^24^) to assess the number of shared and distinct nonzero genetic variants between MDD and another trait required to explain at least 90% of heritability in the two traits, referred to as ‘causal’ variants. As it assesses overlap regardless of the effect direction of each variant, it gives a more adequate picture of local genetic correlations of opposite directions that cancel each other out in the genome-wide genetic correlation estimate. We excluded the *HLA* region, following the software recommendations^24^. To assess the stability of point estimates and estimate their standard deviations, we fitted the MiXeR model 20 different times for 2 million randomly selected SNPs with minor allele frequency of at least 5% (Supplementary Table 6). The number of 20 runs follows recommendations published previously^5^.

The MiXeR model was evaluated for each trait by (1) comparing the Akaike information criterion (AIC) of the univariate MiXeR model with the LDSC model, which does not include a parameter representing polygenicity (Supplementary Table 5); (2) comparing the AIC of the bivariate MiXeR model with the AIC of the model with the least possible amount of polygenic overlap required to explain observed genetic correlation; (3) comparing the AIC of the bivariate MiXeR model with the AIC of the model with maximum amount of polygenic overlap (in such a model, all risk variants of the least polygenic trait are also risk variants of the other trait) and (4) evaluating the stability of the point estimates over the 20 runs (Supplementary Table 6). These metrics have been described in detail previously^5^. Except for the univariate test, the MiXeR model failed these checks for IL6, which was therefore excluded from the results. The MiXeR model performed poorly for the IL6 probably because it is a trait with low polygenicity and was measured in a relatively small sample (Extended Data Fig. 2 and Supplementary Table 1).

To complement the univariate polygenicity analysis in MiXeR, we estimated polygenicity using a Bayesian framework implemented in SBayesS^46^. We used a 15,000-sample Markov chain with a 5,000-sample burn-in. The SBayesS methods did not converge for peripheral artery disease, possibly due to low number of cases in the GWAS.

### Shared liability to MDD and ASCVD

To move beyond bivariate association to multivariate overlap, we conducted factor analysis on MDD and the CVDs using genomic SEM^26^ to assess whether genetic latent factors could explain the genetic covariance between the traits. Genomic SEM uses LDSC to estimate the genetic covariance matrix between traits. It then uses the genetic covariance matrix in a SEM framework to identify multivariate relationships in the data. We set CFI at >0.90, SRMR at <0.03 and standardized factor loadings *β* > 0.3 as criteria for acceptable model fit. The best model fit was found for a factor with coronary artery disease, peripheral artery disease, heart failure and stroke as indicators, which we interpret as a latent genetic factor representing ASCVD. To model a genetic factor for shared liability to MDD and ASCVD, we defined a higher-order factor with this ASCVD factor plus MDD as indicators. The standardized loading of the first indicator (coronary artery disease) was set to 1. The residual variance of the ASCVD factor was forced to be 0, so that all variance was forced into the MDD–ASCVD factor. For comparison, we also estimated a common factor model for ASCVD without MDD (Extended Data Fig. 3a).

We conducted a summary statistics-based GWAS on the MDD–ASCVD second-order latent factor to identify variants associated with this latent shared liability. We used the package-default diagonally weighted least squares estimator. To derive genome-wide significant independent loci we used the plink clumping procedure as implemented in FUMA^76^, with *R* = 0.6 and distance or 250 kb, and reference data from 1000 Genomes. To assess the heterogeneity of the SNP effects, we fit an independent pathway model for each SNP, where each indicator was regressed on the SNP directly instead of forcing the effect through the latent factor. We compare the common pathway ${\chi }_{{\rm{SNP}}}^{2,{\rm{com}}}$ to the independent pathway ${\chi }_{{\rm{SNP}}}^{2,\,{\rm{ind}}}$ to derive the heterogeneity measure *Q*_SNP_. For follow-up analyses, we filtered out all SNPs that had effects that were more consistent with an independent pathway model at ${P}_{{{{Q}}}_{{\rm{SNP}}}}$ <0.05. This stringent procedure filters out SNPs with heterogeneous effects on MDD and the ASCVD factor. This way, variants are excluded that should be considered risk variants for MDD or CVDs separately rather than risk variants for MDD–ASCVD. We did not observe genomic inflation, indicating that results were not strongly affected by population stratification (LDSC intercept of 1.02).

We used FUMA to check whether genome-wide significant SNPs for the MDD–ASCVD factor were enriched in genome-wide significant SNPs for traits in the GWAS catalog. To define unique loci, we overlaid the independent genomic risk loci (now clumped at *R*^2^ = 0.1 and 3,000 kb window) for MDD–ASCVD with the risk loci for the constituent traits (MDD, peripheral artery disease, coronary artery disease, heart failure and stroke). We used the ‘intersect’ function in bedtools to assess whether risk regions were overlapping. If they were independent (according to the clump criteria) they were regarded as novel loci. We also identified genes whose regulation is significantly impacted by the top significant SNPs to interpret the biological implications of our findings. For this, we use eQTL estimates from FUMA based on PsychENCODE reference data^77^. We conducted a GWAS for the latent ASCVD factors using the same procedure (though without filtering out heterogeneous SNPs).

To externally validate the latent MDD–ASCVD GWAS results, we computed PRS using LDpred2 (automatic mode with HapMap3 LD reference data), as implemented in the R package bigsnpr. We compared the MDD–ASCVD PRS with a PRS based on MDD only and with a PRS based on latent CVD without MDD. As a target sample we used the UKB. Summary statistics for ASCVD traits excluding the UKB were unavailable, and we chose to leave the UKB in for all traits. Sample overlap is likely to lead to overfitting, resulting in an inflation of explained variance. However, this is less of a concern when comparing PRS among themselves, rather than assessing absolute predictive value. As target phenotypes, we extracted CVD and MDD cases according to healthcare registry International Classification of Diseases (ICD) codes from data field 41270 (Supplementary Table 14). We used logistic regression analysis to predict case status from each PRS while controlling for the first ten principal components for ancestry, sex and year of birth. Continuous variables were standardized and centered. We used Nagelkerke’s *R*^2^ to estimate explained variance in the disease traits.

### Tissue and cell-type analysis

To gain insight into the biological mechanisms underlying the shared genetic liability to MDD and ASCVD, we performed a tissue and cell-type analysis using partitioned LDSC. Cell-type identification was based on the top decile of specifically expressed genes (referred to as top decile expression proportion (TDEP) genes). The methodology has been described extensively in previous studies^78–80^.

We identified TDEP genes for brain cell types from single-nucleus RNA sequencing data measured in dissections of three adult human postmortem brain samples for the Adult Human Brain Atlas^27^, part of the Human Cell Atlas. We used the manually annotated 31 superclusters and 461 clusters provided by the atlas^28^. We considered a curated set of 18,090 protein-coding autosomal genes, excluding those in the *HLA* region (because the method relies on LDSC), with expression in at least one of the 461 cell clusters.

To establish TDEP genes for 16 human tissues, we utilized bulk RNA sequencing data from GTEx v8 (ref. ^81^). In line with previous research, we removed tissues with <100 donors and nonnatural tissues (for example, cell lines) as well as testis tissues (expression outlier)^78^. Before analysis in partitioned LDSC, we expanded the boundaries of TDEP genes by 100 kb to include possible enhancers or promoters.

We tested the associations between GWAS traits and tissue or cell types by estimating heritability enrichment within the TDEP genes for each tissue or cell type. To ensure that annotation enrichment could not be better explained by other overlapping annotations, we adjusted for enrichment in 53 previously defined baseline LDSC annotations (LDSC v1.0.1) of different types of genomic regions: coding, untranslated, promoter, intronic, enhancer, histone marks and other epigenetic marks^80^.

### Pathways linking MDD and CVDs

#### Adjusted genetic correlation and mediation in genomic SEM

We employed several different techniques to assess whether the association between MDD and CVD could be explained by shared risk factors.

First, we estimated the genetic correlations of MDD with the individual CVD traits as well as the latent ASCVD factor adjusting for the effects of risk factors using genomic SEM. To aid interpretation, we added the covariates in groups, controlling for all trait groups separately (psychosocial or lifestyle, childhood maltreatment, metabolic or inflammation traits; Supplementary Table 1). We only included traits in the covariate groups that showed statistically significant genetic correlation with MDD. We did not adjust for blood pressure traits since none of the blood pressure traits showed statistically significant genetic correlations with MDD. Attenuation of genetic correlation after adjustment was taken to indicate that shared risk factors account for some (or all) of the association between MDD and CVD.

Next, we modeled the risk factors explicitly as mediators in genomic SEM. If the direct effect is attenuated in the mediation model as compared with the covariate model, we view this as tentative support for the existence of mediation (following procedures suggested by the software developers^82^). Note however that this interpretation relies on untestable assumptions. Finally, we also tested the effects of individual risk factors (both as covariates and mediators) instead of grouping them.

#### Univariable and multivariable MR

To assess tentative causal associations between MDD and CVD and risk factors, we used two-sample MR. We assessed the effects of MDD on the CVDs and risk factors, the effect of the CVDs and risk factors on MDD and the effect of latent MDD–ASCVD on risk factors. MR uses genetic variants as instrumental variables to assess the presence of causal effects of an ‘exposure’ on an ‘outcome’. Core assumptions include that the instrumental variables are robustly associated with exposure and are not associated with the outcome (other than through the effect from exposure) or unmeasured confounders. We used the IVW estimate in the two-sample MR R package^83^ as our main estimate. As instrument SNPs, we selected independent GWAS hits at *P* *<* 5 × 10^−8^, *R*^2^< 0.001 and distance <5 Mb. In the analysis of the effect of genetic instruments of peripheral artery disease, physical activity, childhood maltreatment and IL6 on MDD risk, we allowed instruments with higher *P* values to be able to reach a total of ten instruments (*P* *<* 1 × 10^−5^). For the MDD–ASCVD exposure, we used SNPs that showed no significantly heterogeneous effects in the genomic SEM model (*Q*_*P*value_ >0.05, see above), to limit the possibility of pleiotropic effects of this, by nature, heterogeneous instrument.

We performed several sensitivity analyses to test and adjust for violation of MR assumptions. All analyses were Steiger filtered, meaning that all SNPs that explained statistically significantly more variance in the outcome than the exposure were excluded as instruments^84^. Weighted median and mode regression were used to correct for effect size outliers that could represent pleiotropic effects^85^. MR–Egger regression was used to assess pleiotropy (pleiotropy leads to a significant intercept) and correct for it (unless *I*^2^ indicated violation of the NO Measurement Error assumption, in which case we did not report MR–Egger results^86,87^). We detected statistically significant pleiotropy in the analysis of the association between MDD and triglycerides, rendering the IVW estimates uninterpretable. Second, to assess the strength of our instruments, we used Cochran’s *Q* to assess heterogeneity in the SNP effects^88^ and the *F*-statistic to control for weak instrument bias^89^. Third, we performed sensitivity analyses to gauge the effect of sample overlap in the GWASs that were used. Although sample overlap has been suggested to not greatly impact MR results when the source GWASs have a large sample size and the overlap is limited^90^, we wanted to ensure sample overlap did not lead to bias in our findings. We assessed the genetic covariance intercepts for all MDD–trait pairs from the LDSC analyses and observed that most were more than 1 s.d. away from 0, indicating that sample overlap was present (Supplementary Table 2). We repeated the analyses with MDD summary statistics leaving out the UKB sample, which is the sample responsible for most of the overlap, and compared the results. Also, we repeated the analyses using LHC MR^47^, which aims to correct for the presence of unmeasured heritable confounders as well as sample overlap.

We also investigated associations of genetic liability to MDD and CVD adjusted for the effect of risk factors using multivariable MR (MVMR) analyses. The difference with the mediation test in genomic SEM is that the MVMR analysis use instrument variables for MDD instead of all genetic variants, which yields results more in line with a causal interpretation. As MVMR relies on regression analysis, it cannot formally test mediation; instead, the causal estimate is adjusted for the risk factor. To support a directional interpretation, we included only risk factors as mediators that were statistically significantly affected by MDD according to the results from the univariable analysis. For the MVMR analysis, we only derive an IVW estimate. Steiger filtering was performed on the exposure–outcome association. These analyses were not replicated in LHC, which does not accommodate multivariable analyses. For ease of interpretation, we again grouped the mediators and adjusted for all mediators in a group concurrently. We only included a mediator in a group if it was significant in the univariate analysis. Additionally, we performed analyses adjusting for single mediators. We selected instruments for each variable in a model by a clumping step with the same parameters as in the univariate MR case (*P* *<* 5 × 10^−8^, *R*^2^ < 0.001). We then combined instruments for all variables in a model into a single set of instruments and performed another clumping step with the same parameters. These instruments were then aligned to the same effect allele. We estimated the effect of genetic liability to MDD on CVD traits adjusting for covariates using multivariate MR with the MVMR R package^91^.

### Statistics and reproducibility

To ensure robust results, we considered only summary statistics of GWAS involving more than 10,000 cases. We maximized sample sizes available for individual analyses instead of performing replication analyses. In addition, we ensure robust results by triangulating evidence from multiple methods and statistical frameworks. These results rely on GWASs of population samples, which are not randomized. Processing and analysis was performed using the R language for statistical computing (version 4.1.0) and the Python programming language (version 2.7.13).

### Reporting summary

Further information on research design is available in the Nature Portfolio Reporting Summary linked to this article.

### Supplementary information

Reporting Summary

Supplementary Table Excel workbook containing all 20 supplementary tables for the paper.

### Source data

Source Data Fig. 2 Statistical source data.

Source Data Fig. 3 Statistical source data.

Source Data Fig. 4 Statistical source data.

Source Data Fig. 5 Statistical source data.

Source Data Fig. 6 Statistical source data.

Source Data Extended Data Fig. 1 Statistical source data.

Source Data Extended Data Fig. 2 Statistical source data.

Source Data Extended Data Fig. 3 Statistical source data.

Source Data Extended Data Fig. 4 Statistical source data.

Source Data Extended Data Fig. 5 Statistical source data.

Source Data Extended Data Fig. 6 Statistical source data.

Source Data Extended Data Fig. 7 Statistical source data.

Source Data Extended Data Fig. 8 Statistical source data.

Source Data Extended Data Fig. 9 Statistical source data.

Source Data Extended Data Fig. 10 Statistical source data.

## Data Availability

Links to download publicly available published GWAS summary statistics data used as inputs in this study are listed in Supplementary Table 1. Single-nucleus RNA sequencing data in the adult human brain can be found at https://github.com/linnarsson-lab/adult-human-brain. Researchers can request access to the UKB data resources at https://www.ukbiobank.ac.uk/enable-your-research/apply-for-access; data for PRS analysis described in this study were accessed under accession number 22224. Gene expression data from human tissues can be found at https://www.gtexportal.org/home/datasets. Summary statistics for GWAS of the MDD–ASCVD and ASCVD latent factors are available via figshare at 10.6084/m9.figshare.25737537 (ref. ^92^).

## Code availability

Code used for processing and analyzing data in this work can be found at https://github.com/jacobbergstedt/MDDCVD_genetics. Code for singularity containers can be found at https://github.com/comorment.
