# Supplementary material for: Distinct biological signature and modifiable risk factors underlie the comorbidity between major depressive disorder and cardiovascular disease
Source: Nat Cardiovasc Res. 2024 Jun 17;3(6):754–69. doi: 10.1038/s44161-024-00488-y (PMC11182748; doi:10.1038/s44161-024-00488-y)
Supplement: Supplementary file 1 — Reporting Summary [file 44161_2024_488_MOESM1_ESM.pdf]

nature portfolio

Corresponding author(s): Jacob Bergstedt, Fang Fang

Last updated by author(s): Jan 26, 2024

Reporting Summary

Nature Portfolio wishes to improve the reproducibility of the work that we publish. This form provides structure for consistency and transparency in reporting. For further information on Nature Portfolio policies, see our Editorial Policies and the Editorial Policy Checklist.

Statistics

For all statistical analyses, confirm that the following items are present in the figure legend, table legend, main text, or Methods section.

n/a	Confirmed	
☐	☒	The exact sample size (*n*) for each experimental group/condition, given as a discrete number and unit of measurement
☐	☒	A statement on whether measurements were taken from distinct samples or whether the same sample was measured repeatedly
☐	☒	The statistical test(s) used AND whether they are one- or two-sided<br>*Only common tests should be described solely by name; describe more complex techniques in the Methods section.*
☐	☒	A description of all covariates tested
☐	☒	A description of any assumptions or corrections, such as tests of normality and adjustment for multiple comparisons
☐	☒	A full description of the statistical parameters including central tendency (e.g. means) or other basic estimates (e.g. regression coefficient) AND variation (e.g. standard deviation) or associated estimates of uncertainty (e.g. confidence intervals)
☐	☒	For null hypothesis testing, the test statistic (e.g. *F*, *t*, *r*) with confidence intervals, effect sizes, degrees of freedom and *P* value noted<br>*Give P values as exact values whenever suitable.*
☐	☒	For Bayesian analysis, information on the choice of priors and Markov chain Monte Carlo settings
☐	☒	For hierarchical and complex designs, identification of the appropriate level for tests and full reporting of outcomes
☐	☒	Estimates of effect sizes (e.g. Cohen's *d*, Pearson's *r*), indicating how they were calculated

Our web collection on statistics for biologists contains articles on many of the points above.

Software and code

Policy information about availability of computer code

Data collection	R version 4.1.0<br>Bash software: cleansumstats 1.6.0.<br>Full data collection scripts can be found at github.com/jacobbergstedt/github.com/jacobbergstedt/MDDCVD_genetics<br>Container data collection software can be found at https://github.com/comorment<br>Data was processed using TSD and slurm
Data analysis	R version 4.1.0<br>R packages: GenomicSEM 0.0.5; TwoSampleMR 0.5.6; MVMR 0.4; LAVA 0.1.0; bigsnpr1.12.4<br>Python version 2.7.13<br>Python software: ldsc 1.0.1; MiXeR 1.3<br>Bash software: plink 1.9, plink2.0, gctb2.05<br>Full data analysis scripts can be found at github.com/jacobbergstedt/MDDCVD_genetics<br>Container data analysis software can be found at https://github.com/comorment<br>Data was analyzed using the TSD cluster

For manuscripts utilizing custom algorithms or software that are central to the research but not yet described in published literature, software must be made available to editors and reviewers. We strongly encourage code deposition in a community repository (e.g. GitHub). See the Nature Portfolio guidelines for submitting code & software for further information.

Data

Policy information about availability of data

All manuscripts must include a data availability statement. This statement should provide the following information, where applicable:

- Accession codes, unique identifiers, or web links for publicly available datasets
- A description of any restrictions on data availability
- For clinical datasets or third party data, please ensure that the statement adheres to our policy

Links to download publicly available published GWAS summary statistics data used as inputs in this study are listed in Supplementary Table 1. Single nucleus RNA sequencing data in the adult human brain can be found at https://github.com/linnarsson-lab/adult-human-brain. Researchers can request access to the UK-Biobank data resources at https://www.ukbiobank.ac.uk/enable-your-research/apply-for-access; data for PRS analysis described in this study were accessed under accession number 22224. Gene expression data from human tissues can be found at https://www.gtexportal.org/home/datasets. Summary statistics for GWAS of the MDD-ASCVD and ASCVD latent factors are available at https://doi.org/10.6084/m9.figshare.25737537.

Research involving human participants, their data, or biological material

Policy information about studies with human participants or human data. See also policy information about sex, gender (identity/presentation), and sexual orientation and race, ethnicity and racism.

Reporting on sex and gender	We adjusted on sex in the PRS analysis. Further consideration of sex is out of scope for our research aims in this project.
Reporting on race, ethnicity, or other socially relevant groupings	We did not consider race, ethnicity, or other socially relevant groupings in this study.
Population characteristics	The UK Biobank is a prospective cohort study decsribed in Bycroft et al. 2018 Nature. Average age for participants at assessment was 58 years; 54.2% are female; and 94.7% are of European ancestry. The populations for the summary statistics GWAS were all of European ancestry. For more detailed information please see publications listed in Supplementary Table 1.
Recruitment	See Bycroft et al. 2018 Nature and publications listed in Supplementary Table 1
Ethics oversight	Ethics approval for the UK Biobank study was obtained from the North West Centre for Research Ethics Committee (11/NW/0382). The work described here was approved by UK Biobank under application number 22224

Note that full information on the approval of the study protocol must also be provided in the manuscript.

Field-specific reporting

Please select the one below that is the best fit for your research. If you are not sure, read the appropriate sections before making your selection.

☒ Life sciences ☐ Behavioural & social sciences ☐ Ecological, evolutionary & environmental sciences

For a reference copy of the document with all sections, see nature.com/documents/nr-reporting-summary-flat.pdf

Life sciences study design

All studies must disclose on these points even when the disclosure is negative.

Sample size	We did not consider summary statistics from GWASs with less than 10,000 cases. We selected broad CVD diseases with large-scale GWASs available at the time of the study. For risk factors, we picked risk factors based on our hypothesis that immunometabolic factors are linked to MDD-CVD comorbidity, combined with availability of GWASs of sufficient sample size and quality.
Data exclusions	Only GWAS based on populations of European ancestry were considered to ensure comparable LD patterns underlying GWAS summary statistics.
Replication	LAVA and MiXeR analyses are complementary and show similar patterns. MDDCVD summary statistics were validated in UK Biobank. For the single nucleus brain transcriptomics, the dataset from Siletti et al. (2022) Science is to the best of the author's knowledge the first and only single-cell transcriptomic census of the entire human brain. For Mendelian randomization we conducted three analyses to ensure robust results the main version, the version without UKB in the MDD summary statistics, and a version using a complementary method latent heritable confounder Mendelian randomization. All conclusions were confirmed across sensitivity analyses and triangulation.
Randomization	We did not employ any experimental batches or groups.
Blinding	We did not do any group allocation for this study.

Reporting for specific materials, systems and methods

We require information from authors about some types of materials, experimental systems and methods used in many studies. Here, indicate whether each material, system or method listed is relevant to your study. If you are not sure if a list item applies to your research, read the appropriate section before selecting a response.

Materials & experimental systems

n/a	Involved in the study
☒	☐ Antibodies
☒	☐ Eukaryotic cell lines
☒	☐ Palaeontology and archaeology
☒	☐ Animals and other organisms
☒	☐ Clinical data
☒	☐ Dual use research of concern
☒	☐ Plants

Methods

n/a	Involved in the study
☒	☐ ChIP-seq
☒	☐ Flow cytometry
☒	☐ MRI-based neuroimaging

Plants

Seed stocks	n/a
Novel plant genotypes	n/a
Authentication	n/a
